# Endothelial cell death after ionizing radiation does not impair vascular structure in mouse tumor models

**DOI:** 10.15252/embr.202153221

**Published:** 2022-07-18

**Authors:** Jakob R Kaeppler, Jianzhou Chen, Mario Buono, Jenny Vermeer, Pavitra Kannan, Wei‐Chen Cheng, Dimitrios Voukantsis, James M Thompson, Mark A Hill, Danny Allen, Ana Gomes, Veerle Kersemans, Paul Kinchesh, Sean Smart, Francesca Buffa, Claus Nerlov, Ruth J Muschel, Bostjan Markelc

**Affiliations:** ^1^ Cancer Research UK and MRC Oxford Institute for Radiation Oncology, Department of Oncology University of Oxford Oxford UK; ^2^ MRC Molecular Hematology Unit, MRC Weatherall Institute of Molecular Medicine, John Radcliffe Hospital University of Oxford Oxford UK; ^3^ In Vivo Imaging The Francis Crick Institute London UK; ^4^ Present address: Department of Experimental Oncology Institute of Oncology Ljubljana Ljubljana Slovenia

**Keywords:** apoptosis, intravital imaging, radiotherapy, tumor endothelial cells, tumor vasculature, Autophagy & Cell Death, Cancer, Vascular Biology & Angiogenesis

## Abstract

The effect of radiation therapy on tumor vasculature has long been a subject of debate. Increased oxygenation and perfusion have been documented during radiation therapy. Conversely, apoptosis of endothelial cells in irradiated tumors has been proposed as a major contributor to tumor control. To examine these contradictions, we use multiphoton microscopy in two murine tumor models: MC38, a highly vascularized, and B16F10, a moderately vascularized model, grown in transgenic mice with tdTomato‐labeled endothelium before and after a single (15 Gy) or fractionated (5 × 3 Gy) dose of radiation. Unexpectedly, even these high doses lead to little structural change of the perfused vasculature. Conversely, non‐perfused vessels and blind ends are substantially impaired after radiation accompanied by apoptosis and reduced proliferation of their endothelium. RNAseq analysis of tumor endothelial cells confirms the modification of gene expression in apoptotic and cell cycle regulation pathways after irradiation. Therefore, we conclude that apoptosis of tumor endothelial cells after radiation does not impair vascular structure.

## Graphical abstract

Radiation of murine tumors using either single (15 Gy) or fractionated (5 × 3 Gy) doses fails to result in significant structural changes of the perfused tumor vasculature. Because apoptosis mainly occurs in non‐perfused vessels and blind ends, the main structural network remains.

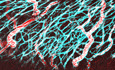

## Introduction

Radiation therapy for cancer results in irradiation of both the cancer cells and the tumor stromal cells including the vasculature. The consequence of radiation to the vasculature is a subject of considerable dispute. At doses higher than the individual doses used in routine radiation therapy treatment for cancer, tumor endothelium undergoes cell death (Castle & Kirsch, 2019). Yet, the contributions of endothelial cell death to the vascular structure are unclear. Neither is its influence on tumor response to radiotherapy. Better understanding of how irradiation modulates the tumor vasculature could provide important insights for the therapeutic regimen design of radiotherapy.

A number of studies have examined the direct death of endothelium resulting from its exposure to irradiation. Sensitization of the endothelium to radiation by means of implantation of xenografts in mice with defects in DNA repair (SCID or DNA‐PK defective) did not reduce the dose of radiation needed for tumor eradication (Budach *et al*, 1993; Garcia‐Barros *et al*, 2010). To precisely focus upon the endothelium, Moding *et al* used a conditional knockout mouse model, in which genetic elimination of ataxia telangiectasia mutated (ATM) was direct only to the endothelium. ATM is required for the repair of radiation‐induced DNA damage as well as cell cycle arrest. Tumors grown in mice lacking ATM in their endothelium were more sensitive to radiation with doses of 20 Gray (Gy) or 3Gy × 10 fractions (F) than tumors growing in their wild‐type counterparts. However, their responses to SBRT radiation with doses of 50 Gy or 4x 20 Gy did not differ significantly from the responses in their wild‐type counterparts (Moding *et al*, 2014, 2015). Genetic elimination of both apoptosis controlling genes Bak and Bax in endothelium did not alter the radiation response of tumors (Moding *et al*, 2015). In both studies by Moding *et al*, an increase in endothelial cell death was not found within the first 24 h after irradiation, though it did occur at later time. In contrast, other studies have focused upon the importance of endothelial death for the determination of tumor response to radiation with differing conclusions. Garcia‐Barros *et al* described an induction of apoptosis in endothelial cells in irradiated tumors peaking at 6 h after irradiation. They also noted a reduced response to radiation in acid sphingomyelinase‐deficient mice, which correlated with a diminished capacity for endothelial apoptosis leading them to propose that early apoptosis of the endothelium itself was a major determinant in tumor response to radiation (Garcia‐Barros *et al*, 2003). None of these studies examined the effects on vascular structure.

The radiation response of tumors and their vasculature is highly time‐ and dose‐dependent. Even within the same tumor type and at different points in the same tumor, the response can be heterogeneous in the scale of the response and its timeline (Dewhirst *et al*, 1990; Hu *et al*, 2016). In murine tumors, single doses of radiation result in considerable, but transient vasodilation peaking at approximately 24 h after irradiation, which is partly attributed to the release of nitrogen oxides (NO); however, this timeline may vary with tumor type (Sonveaux *et al*, 2003; Li *et al*, 2007; Hu *et al*, 2016). Whether this pattern persists after each dose of fractionated radiation is not clear. The perfusion of tumors in some murine models diminishes at later times over days, depending on the doses of radiation. With single doses varying from 5 to 20 Gy, various researchers have found reduced perfusion of the tumor while others have found no change (for comprehensive reviews see Park *et al*, 2012; Kim *et al*, 2015) and in many cases there was evidence of vascular death, leakiness of blood vessels and lack of perfusion in parts of the tumor. Lower doses have much smaller effect on vascular function (Kioi *et al*, 2010; Maeda *et al*, 2012; Moding *et al*, 2013; Brown *et al*, 2014; Demidov *et al*, 2018). With fractionated radiation, less vascular damage was reported in murine models (Potiron *et al*, 2013).

Clinical studies, in contrast, have emphasized reoxygenation and increased perfusion after radiation. While reoxygenation in murine models has been seen within hours and persists for 48 h, clinical studies have generally looked substantially later after radiation (Moeller *et al*, 2004; Song *et al*, 2016). In a study examining hypoxia with [^18^F]‐MISO PET before, during, and after treatment of head and neck cancer tumors, hypoxia was reduced midcourse in some patients and at the end of radiation therapy treatments, while others, approximately half, had no change. Those patients with persistent hypoxia had poorer outcomes, suggesting that patients with persistent tumor hypoxia might benefit from more intensive treatments (Zips *et al*, 2012; Lock *et al*, 2017, 2019). One possible explanation for tumor hypoxia is reduced perfusion, although other explanations could account for increased oxygenation (Good & Harrington, 2013) including changes in the oxygen consumption rate (Clement *et al*, 1978; Ashton *et al*, 2016; Gallez *et al*, 2017). Some tumors were found to have higher levels of perfusion at intermediate times in a course of radiation therapy, but in other cases perfusion remained unchanged for at least several weeks in both cervical and in head and neck cancers (Shibuya *et al*, 2011; Lock *et al*, 2017). The increased perfusion in Shibuya *et al* and reduced hypoxia in Lock *et al* were both associated with improved patient outcome (Shibuya *et al*, 2011; Lock *et al*, 2017). However, at the end of a full course of fractionated radiation, perfusion was often decreased.

These studies raise the question of how the vascular structure is altered by radiation. To address this question, we used video 2‐photon microscopy of murine tumors receiving radiation with repeated observations of the same tumor. Many of the studies examining tumor vasculature that used intravital video microscopy of tumors have relied upon infusion of fluorescent dyes to visualize vessels, yet this method will only visualize perfused vessels (Moeller *et al*, 2004; Maeda *et al*, 2012). More recently, optical coherence tomography has been used to that end (Li *et al*, 2007; Demidov *et al*, 2018). Genetically engineered mouse models (GEMM) mice with fluorescent endothelial cells have been used to follow tumor vascular progression (Mathivet *et al*, 2017; Stanchi *et al*, 2019). We used transgenic mice bearing a Cre recombinase‐tamoxifen receptor fusion protein (Cre‐ERT2) driven by the VE‐cadherin promoter (Monvoisin *et al*, 2006) combined with a floxed‐stop cassette upstream of tdTomato gene (henceforth VE‐TOM mice). Treatment of these mice with tamoxifen results in endothelial cells tagged with tdTomato. Using these mice, the overwhelming majority of endothelial cells are fluorescently labeled, so that both functional perfused vessels and sprouts, blind ends, and other non‐perfused vessels could be imaged. Perfused vessels can be distinguished as those labeled with infused fluorescent dyes.

To observe the effect of radiation on tumor vasculature and endothelium, we used these methods to image tumors in non‐orthotopic abdominal window chambers after radiation. We found little disruption in functional vascular structure despite the death of endothelial cells after 15 Gy of irradiation. However, death of endothelial cells was mainly in smaller, non‐perfused vessels explaining why this has little effect on the overall vascular network after radiation. For the remaining endothelial cells, irradiation resulted in cell cycle arrest allowing remodeling of the existing vasculature, without significant impairment in vascular function. In tumors with a substantial proportion of smaller non‐perfused vessels, the loss of endothelial cells resulted in increased average vascular diameter overall and longer inter‐branch distances leading to potentially more effective vascular structures evolving in the tumor from the preexisting vessels.

These results begin to reconcile the observation of endothelial cell death with only minor changes in functional vascular structure.

## Results

### Fluorescent labeling of the tumor vasculature

**Figure 1:**
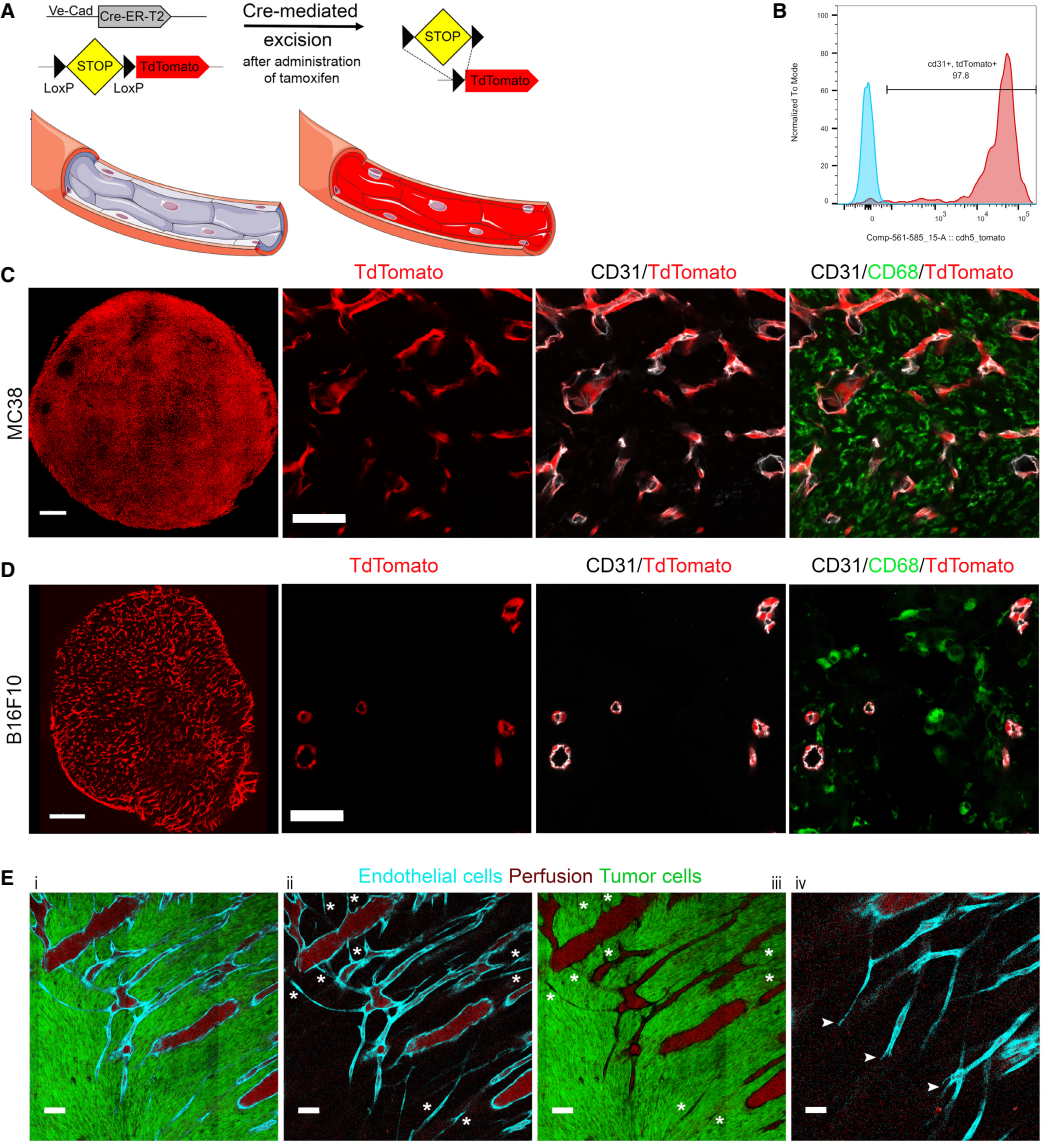
tdTomato expression in ECs and TECs in VE‐TOM mice ASchematic of the Ve‐Cad (Cre‐ERT2) system. Administration of tamoxifen by gavaging in adult VE‐TOM mice activates the Cre‐LoxP system in endothelial cells inducing tdTomato expression.BFACS analysis histogram of TECs from MC38 tumors 30 days after tamoxifen administration. Percent of tdTomato‐positive CD31‐positive TECs (red) and control TECs (blue).CRepresentative low‐power image of a MC38 tumor vasculature (left), TECs expressing tdTomato (red). High‐power images (right) of the same tumor: tdTomato (red) in TECs, co‐stained for CD31 (white) and CD68 (green).DRepresentative low‐power image of a B16F10 tumor vasculature (left), TECs expressing tdTomato (red). High‐power images (right) of the same tumor: tdTomato (red) in TECs, co‐stained for CD31 (white) and CD68 (green).ERepresentative image (Ei) of a MC38 tumor. GFP‐positive tumor cells (green), TECs (cyan), and infused Qdots (red) indicating perfused vessels. (Eii and Eiii) Asterisks, non‐perfused tumor vessels, (Eiv) arrowheads, tumor vessel sprouts. Data information: Scale bar in (C and D): 1 mm and 50 μm, respectively; in (E): 100 μm.

**Figure EV1:**
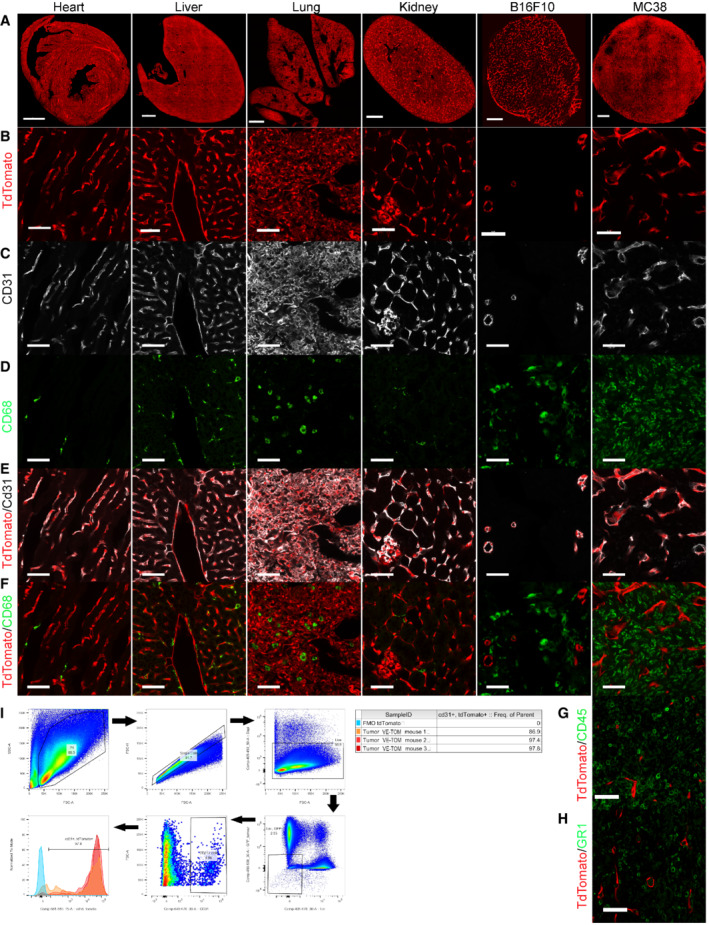
Evaluation of tdTomato‐positive TECs in VE‐TOM mice ARepresentative low‐power microscopic images (top row) of vasculature from tissue sections of organs and tumors expressing tdTomato (red) in endothelial cells.BRepresentative high‐power microscopic images of the same tissue section expressing tdTomato.C, DSamples are stained for CD31 (white, C) and CD68 (green, D).E, FMerged channels of B + C (E) and B + D (F).GRepresentative high‐power microscopic image of tdTomato (red) and CD45 (green).HRepresentative high‐power microscopic image of tdTomato (red) and GR1 (green).IFlow cytometry, gating strategy, and histogram of MC38 tumor TECs 30 days after tamoxifen administration. Percent of tdTomato‐positive CD31‐positive TECs (red) and FMO (blue). Data information: Scale bar in (A); 1 mm; scale bars in (B–H): 50 μm.

In order to visualize the response of the tumor vasculature to irradiation, we used a transgenic mouse model in which the fluorescent protein tdTomato is expressed in both normal and tumor endothelial cells (EC). As described by Wang *et al* (2010), transgenic mice (Tg(Cdh5‐cre/ERT2)1Rha) bearing Ve‐CadherinCreERt2 express an estrogen receptor‐responsive Cre in VE‐Cadherin‐positive cells, which are predominantly ECs. These mice were crossed with Gt(ROSA)26Sortm9(CAG‐tdTomato)Hze mice so that activation of Cre by tamoxifen resulted in EC expression of tdTomato (schematic shown in Fig 1A). The expression of tdTomato in vascular endothelium was confirmed in all organs examined (Fig EV1A–H). Similarly, tumor ECs (TEC) identified as CD31‐positive cells in allografted tumors, both from MC38, a highly vascular tumor (colon adenocarcinoma) and the less vascular B16F10 (melanoma), were rendered generally over 90% tdTomato positive (Figs 1B–D and EV1I). Using prior selection of mice with greater than 95% fluorescent EC (based upon imaging described in Methods section), we used intravenous injection of Qdots to distinguish perfused from non‐perfused tumor vessels, that is, vessels labeled with the infused Qdots and vessels not labeled with it. Smaller diameter vessels were more likely to be non‐perfused (Fig 1E). In an analogous murine tumor model, Roodhart et al. described a subset of bone marrow‐derived monocytes‐positive cells (BMDCs) lining tumor blood vessels that were also fluorescent after tamoxifen treatment and this subset was reported to increase after treatment of the mice with chemotherapy (Roodhart *et al*, 2013). We, therefore, examined control and irradiated MC38 and B16F10 tumors and organs in naïve tdTomato endothelial‐labeled mice for cells positive for CD68 (Fig EV1D and F), CD45 (Fig EV1G), GR1 (Fig EV1H), and tdTomato. High‐resolution microscopy images of thick sections of MC38 and B16F10 naive and irradiated tumors did not reveal tdTomato‐positive cells also expressing CD45, GR1, or CD68 (Figs 1C and D, and EV1F–H). Thus, by showing that these mice have robust tdTomato labeling of ECs which is absent in BMDCs, we have developed the conditions to apply intravital microscopy of tumors generated in mice to investigate the development of the tumor vasculature and examine its response to radiation.

**Figure 2:**
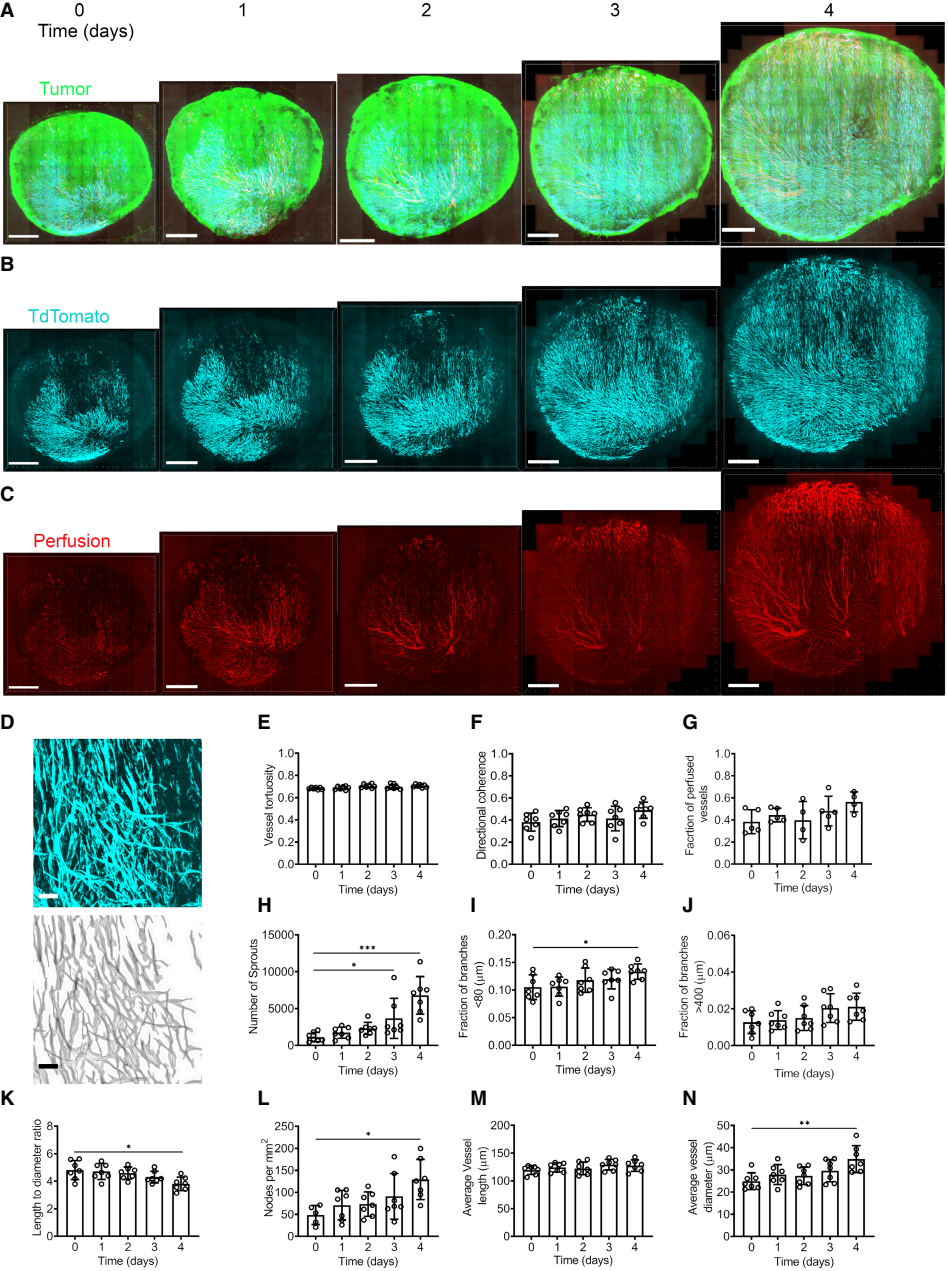
Daily‐serial intravital microscopy of MC38 tumors in VE‐TOM mice A–CThe developing tumor vasculature was imaged over time in an abdominal window chamber model with two‐photon microscopy with (A) GFP‐labeled tumor cells, (B) TdTomato‐labeled TECs in cyan, and (C) perfusion.DTop: a representative image of MC38 tumor vasculature, bottom: segmentation with skeletonization.E–NThe following parameters were quantified from the segmented image each day: (E) vessel tortuosity, (F) directional coherence, (G) perfusion, (H) number of sprouts,(I) fraction of branches <80 μm, (J) fraction of branches >400 μm, (K) length‐to‐diameter ratio, (L) nodes per mm^2^, (M) vessel length, and (N) vessel diameter. Error bar represents mean ± SD (*n* = 4–7 biological replicates); *P < 0.05, **P < 0.01, ***P < 0.0001 by one‐way analysis of variance with multiple comparisons (ANOVA). Data information: Scale bar: 1 mm in (A–C) and 100 μm in (D).

### Daily‐serial intravital microscopy of tumors in Ve‐Cadherin‐CreERt2‐tdTomato (VE‐TOM) mice

**Figure EV2:**
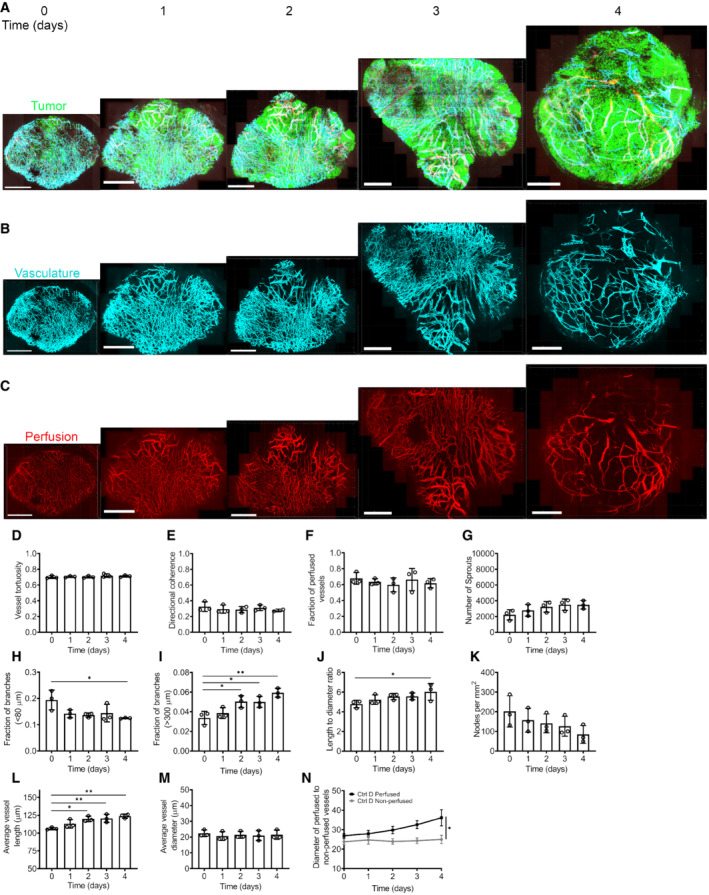
Time‐lapse intravital microscopy of B16F10 tumor in VE‐TOM mice A–CThe developing tumor vasculature was imaged over time in an abdominal window chamber model with two‐photon microscopy with (A) GFP‐labeled tumor cells, (B) TdTomato‐labeled TECs in cyan, and (C) perfusion.D–MThe following parameters were quantified from the segmented image each day: (D) vessel tortuosity, (E) directional coherence, (F) perfusion, (G) number of sprouts, (H) fraction of branches <80 μm, (I) fraction of branches >400 μm, (J) length‐to‐diameter ratio, (K) nodes per mm^2^, (L) vessel length, and (M) vessel diameter.NAverage diameter of perfused to non‐perfused vessel in MC38 tumors. Data information: Data represent mean ± SD, *n* = 3 biological replicates; *P < 0.05, **P < 0.01 by one‐way analysis of variance with multiple comparisons (ANOVA). Scale bar in (A), (B), and (C): 1 mm.

To observe tumor vascular development, we injected GFP‐labeled MC38 or B16F10 cells into an abdominal window chamber in VE‐TOM mice and imaged the developing tumor vasculature over time (Figs 2A–C and EV2A–C). We segmented the images obtained with a previously published algorithm and used the segmented image to compute various vascular network parameters (Fig 2D) (Bates *et al*, 2017, 2019). Some parameters remained unchanged with time such as tortuosity, directional coherence, and the fraction of perfused vessels (Figs 2E–G and EV2D–F). MC38 showed a substantial increase in sprout numbers as the tumors grew in size, and although the number of sprouts in B16F10 tumors increased, it was by a lesser amount (Figs 2H and EV2G). The highly vascular MC38 had a greater fraction of vessels with frequent branching (fraction of vessels with branches separated by <80 μm) than the less vascular B16F10 (Figs 2I and EV2H). At the same time, there was no difference in the fraction of vessels with larger distances between branches in MC38 resulting in a decreased length between branches to diameter ratio (Fig 2J and K). The opposite was seen in B16F10 (Fig EV2H–J), resulting in an increase in the length‐to‐diameter ratio (Fig EV2J). These differences are consistent with MC38 being the more highly vascular tumor (Fig 2L) than B16F10 (Fig EV2K). The average vessel length and diameter showed different changes during tumor growth between the two tumor types as it was increasing in time in the MC38 tumors (Fig 2M and N) whereas it did not change in B16F10 tumors (Fig EV2L and M). However, in both cases, the majority of non‐perfused vessels had diameters below 25 μm (Fig EV2N). It should be noted that little leakage of Qdots was not observed in either model (Figs 2C and EV2C).

### Effect of irradiation on tumor vascular network properties

**Figure 3:**
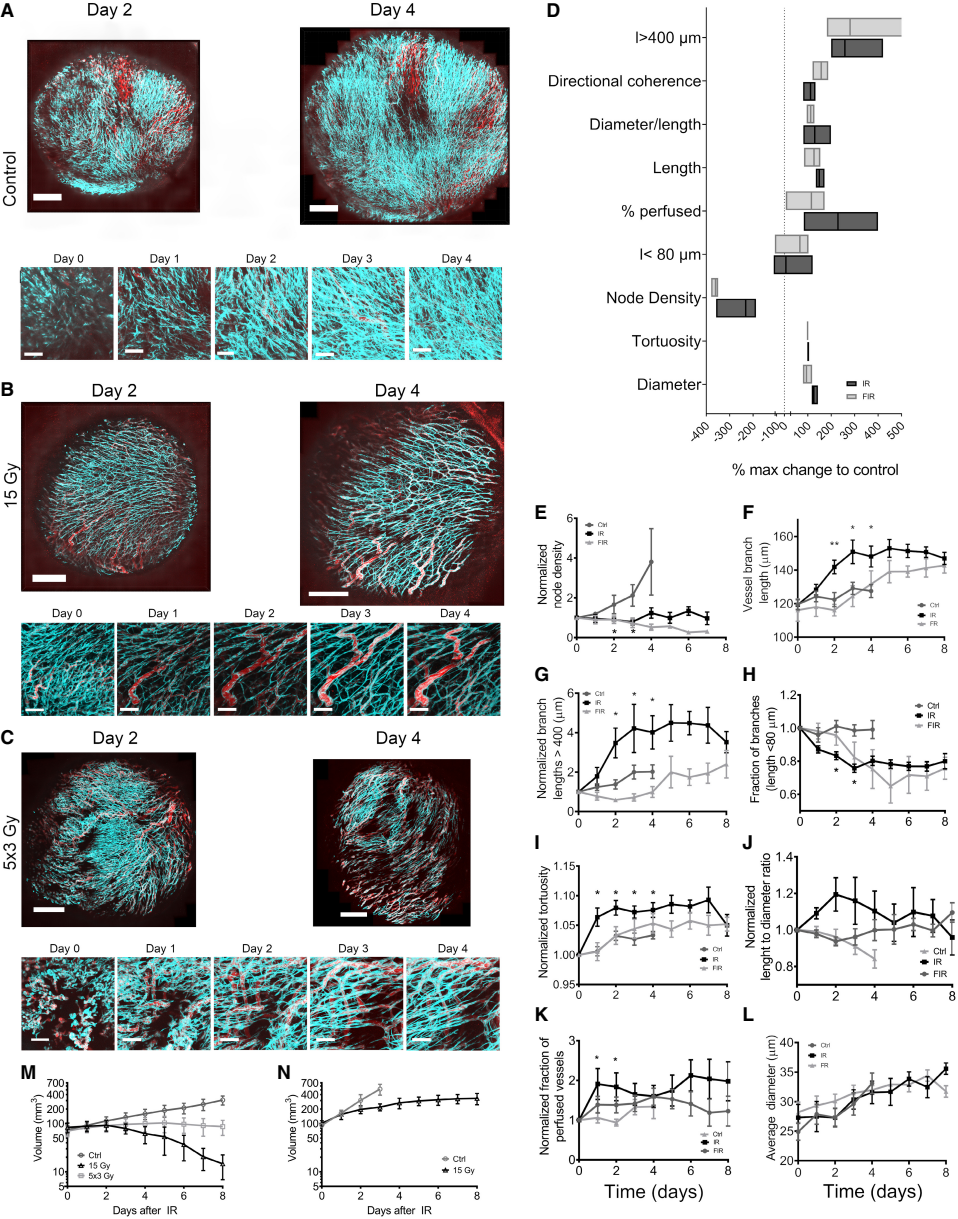
Daily‐serial intravital microscopy of MC38 tumors in VE‐TOM mice after irradiation The developing MC38 tumor vasculature was imaged in an abdominal window chamber model with two‐photon microscopy after IR at the indicated times.
A(top) Representative two‐photon images of a control MC38 tumor vasculature (cyan) and perfusion (red) at day 2 and 4. (Bottom) zoom of the same tumor region from day 0 to day 4 of the tumor vasculature (cyan) and perfusion (red).B, CSingle dose of 15 Gy IR (B) and 5 × 3 Gy (C) fractionated IR‐treated MC38 tumor vasculature (top) at day 2 and day 4. (Bottom) zoom of the same tumor region from day 0 to day 4 of the tumor vasculature (cyan) and perfusion (red).DThe largest change from controls for each parameter after single Gy and fractionated 5 × 3 Gy IR. Floating bars represent min‐max values, and the central line represents the mean value.E–LQuantified vascular parameters, for each day of imaging the following parameters, were quantified from segmented images and normalized to day one if indicated: (E) normalized node density, (F) normalized tortuosity, (G) normalized branch length < 80 μm, (H) normalized branch length > 400 μm, (I) vessel branch length, (J) vessel diameter, (K) normalized length‐to‐diameter ratio, (L) normalized perfused vessels.M, NSubcutaneous tumor growth measurements of MC38 (M) and B16F10 (N) tumors. Data information: Error bar represents mean ± SEM (*n* = 4–7 biological replicates per group in (D–L), *n* = 11–12 biological replicates per group in (M), and *n* = 7–9 biological replicates per group in N); *P < 0.05, **P < 0.01 by analysis of variance (ANOVA). Scale bar: in (A–C) 1 mm (top) and 250 μm (bottom), respectively.

**Figure EV3:**
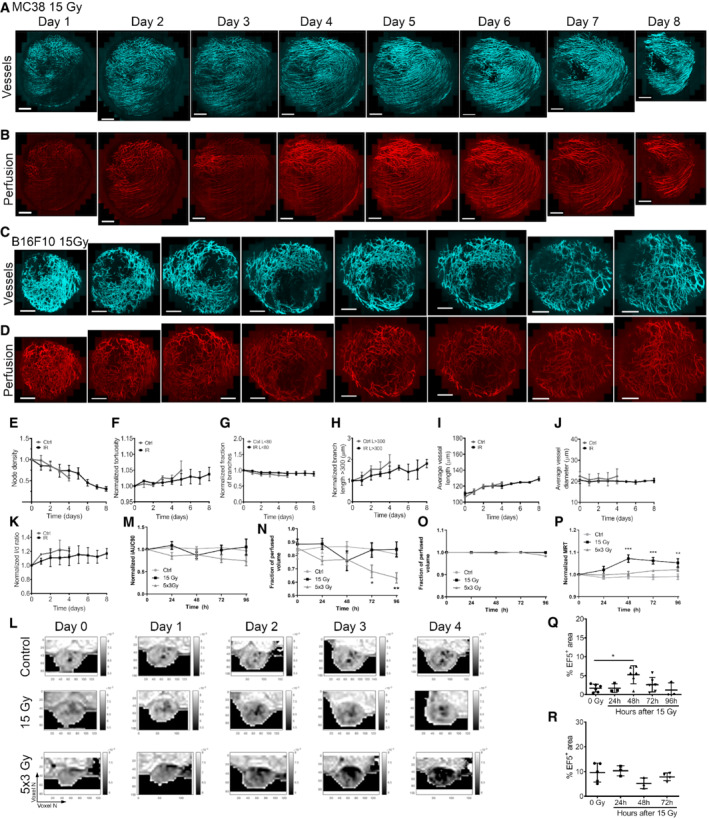
Time‐lapse intravital microscopy of MC38 and B16F10 tumors of VE‐TOM mice, DCE‐MRI imaging of MC38 tumors and EF5 analysis in MC38 and B16F10 tumors A, BTimeline of MC38 tumor vasculature (cyan, A) and perfusion (red, B) after single dose of 15 Gy.C, DTimeline of B16F10 tumor vasculature (cyan, C) and perfusion (red, D) after single dose of 15 Gy IR.E–KQuantified B16F10 tumor vascular parameters, for each day of imaging the following parameters were quantified from segmented images and normalized to day one if indicated: (E) normalized node density, (F) normalized tortuosity, (G) normalized branch length < 80 μm, (H) normalized branch length > 300 μm, (I) vessel branch length, (J) vessel diameter, and (K) normalized length‐to‐diameter ratio.L–PDCE‐MRI imaging and quantification of subcutaneous MC38 tumors. Representative DCE‐MRI images of subcutaneous MC38 tumors injected with gadolinium‐based contrast agent (L). Initial area under the curve (first 90 s after contrast agent injection) iAUC90 was quantified and normalized to day 0 (M). Fraction of perfused tumor volume at 90 s after contrast agent injection (N). Fraction of perfused tumor volume at the end of the imaging session (O). Mean residence time of the contrast agent (P).Q, RQuantification of percent of EF5^+^ tumor area in MC38 tumors (Q) and B16F10 tumors (R) after irradiation. Data information: Data represent mean ± SEM, *n* = 3–4 biological replicates in (E–K), *n* = 6–7 biological replicates in (M–P), *n* = 3–6 biological replicates in (Q, R). *P < 0.05, **P < 0.01 by analysis of variance (ANOVA). Scale bars: in (A–D) 1 mm. Color scale in (L) represents the signal enhancement in DCE‐MRI images due to the presence of gadolinium‐based contrast agent.

We next asked what effect single and fractionated irradiation had on the tumor vasculature. Both MC38 and B16F10 tumors were irradiated with a single dose of 15 Gy, and a group of MC38 tumors also received fractionated radiation of five doses of 3 Gy delivered daily over five consecutive days. MC38 tumors regressed after 15 Gy and had a growth delay with fractionated IR. B16F10 tumors had a growth delay with 15 Gy, which was less pronounced than that in MC38. We used the same dose with different tumor responses so that the endothelium would receive the same dose in each case. The tumor vascular response was followed by imaging tumors in the window chamber (Figs 3A–C and EV3A–D). Figure EV3A–D shows representative MC38 and B16F10 tumors and their vasculature response over the first 7 days after IR. The control tumor as shown earlier and in Fig 3A has gained substantially in size with persistence of the initial vasculature and new angiogenesis (Fig 2A–C). After a single dose of 15 Gy, many of the smaller, poorly perfused vessels have been eliminated, but the more substantial, perfused vessels mainly remained intact (Fig 3B). This was evident by a decrease in node density (Fig 3D and E) (a measure of branching density) and an overall increased distance between vessel branching points (Fig 3D and F) compared with the un‐irradiated control and a two to threefold increase in the percentage of large vessels with a distance between branch points greater than 400 μm (Fig 3G) and a loss of smaller vessels (lengths between branches <80 μm), which are also more likely to be non‐perfused (Fig 3H). We measured a decrease in vessel tortuosity (Fig 3I), an increase in the length‐to‐diameter ratio (Fig 3J), and an increase in the fraction of perfused vessels between 24 and 48 h after a single dose of 15 Gy (Fig 3K). After fractionated radiation, the most prominent change was a decrease in node density (Fig 3E), although there was a decrease in the fraction of highly branched vessels (< 80 μm), over a longer time (Fig 3H). Thus, fewer perturbations were seen in the vascular structure than after a single dose (Fig 3D). Parameters such as perfused fraction of vessels, the length‐to‐diameter ratio, and tortuosity exhibited small or no changes after fractionated IR (Fig 3I–K). Neither single dose of 15 Gy nor fractionated radiation affected the average diameter of blood vessels (Fig 3L). These results suggest that the numbers of highly branched vessels (Fig 3F) and the generation of new vessels (Fig 3E) are reduced by IR, but to a substantially lesser extent after fractionated IR. No indication of vascular rupture based on leakage of Qdots was observed (Fig 3A–C), and no other evidence of compromise of functional vascular structure was evident (Fig EV3A and B). In contrast, after irradiation of B16F10 tumors, which have fewer smaller or non‐perfused vessels, we found no changes in our measured vascular parameters, after a single dose of 15 Gy (Fig EV3E–K). Lastly, the tumor growth response to IR is shown in Fig 3M and N.

**Figure EV4:**
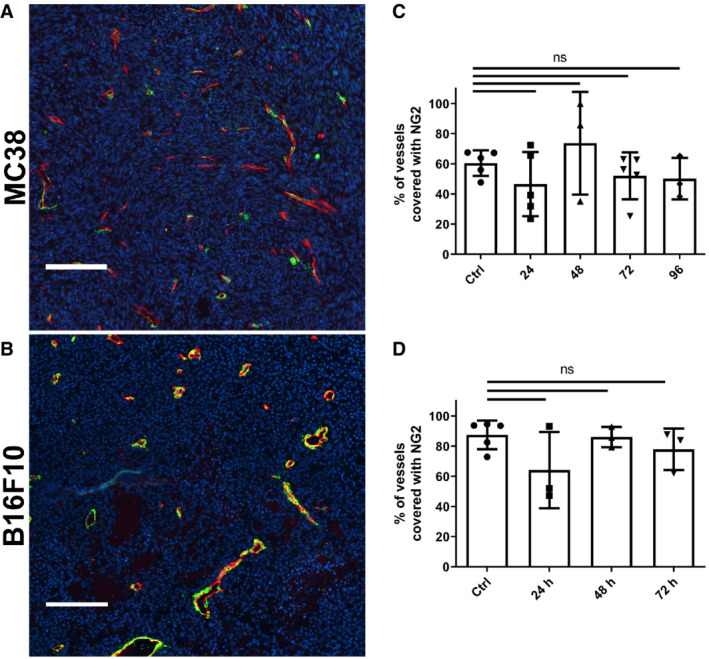
Co‐staining of tumor blood vessels with NG2 and CD31 A, BRepresentative immunofluorescent micrographs of control and treated tumor vasculature from (A) MC38 tumors and (B) B16F10 tumors stained with anti‐CD31 (red), anti‐NG2 (green) and counterstained with Hoechst (blue).C, DQuantification of vessel coverage with NG2 after single dose of 15 Gy IR from immunofluorescent images of whole (C) MC38 tumor (*n* = 3–5 biological replicates per group) or (D) B16F10 tumor sections (*n* = 3–5 biological replicates per group). Data information: Scale bar: 100 μm. Data represent mean ± SD. ns—*P* > 0.05 by one‐way analysis of variance with multiple comparisons (ANOVA).

We also failed to find any decrease in fraction of perfused vessels in either model; however, we observed a transient increase in the percentage of perfused vessels in the MC38 model after a single dose of radiation (Fig 3I). In contrast, we observed that the fraction of initially perfused tumor volume measured by DCE‐MRI has slightly, but not statistically significantly decreased 48 h after single dose of 15 Gy, but returned to pretreatment values within 72 h after irradiation (Fig EV3M). This was also observed with EF5 staining, indicating a slight increase in percent of hypoxic tumor region at 48 h after single dose of 15 Gy (Fig EV3Q). On the contrary, in the B16F10 tumors, there was even a slight non‐significant decrease in the percent of EF5‐positive hypoxic tumor at 48 h after a single dose of 15 Gy (Fig EV3R). When tumors were irradiated with fractionated 5 × 3 Gy irradiation, the initial fraction of perfused tumor volume did not return to pretreatment values, but even decreased during the observation period (Fig EV3N). We further evaluated the initial area under the curve (iAUC), fraction of perfused tumor volume at the end of the DCE‐MRI imaging, and mean resident time (MRT). The only parameter that was changed by irradiation was MRT, which increased after a single dose of 15 Gy at 2–4 days (Fig EV3L–P). Lastly, we have co‐stained tumor section for NG2 proteoglycan and CD31 to determine whether irradiation affects the pericyte coverage of tumor blood vessels; however, we did not observe such effects in either the MC38 or the B16F10 tumor model (Fig EV4A–D). Thus, despite a dose of radiation that has been reported to lead to extensive apoptotic endothelial death, we failed to detect any changes in vascular structure that could compromise vascular function.

### Tumor endothelial cell death and proliferation after irradiation

**Figure 4:**
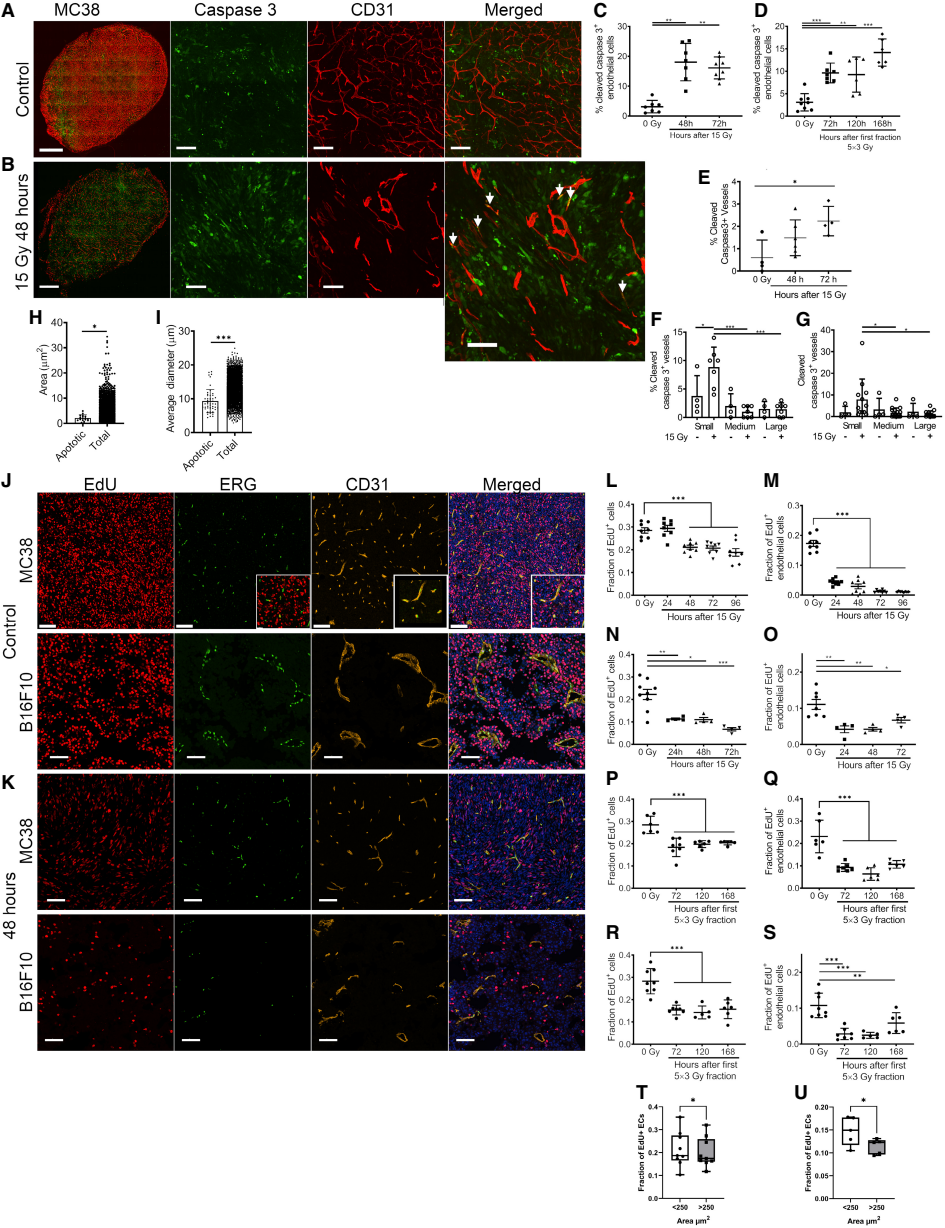
TEC apoptosis and proliferation after single and fractionated IR A, BRepresentative immunofluorescent micrographs of control and treated tumor vasculature from MC38 tumors. Tumor vessels (anti‐CD31) and apoptosis (anti‐cleaved caspase‐3) in (A) control and (B) 48 h after 15 Gy IR. Arrows indicate apoptotic TEC.Cquantification of apoptotic vessels after single dose of 15 Gy IR from immunofluorescent images of whole MC38 tumor section (*n* = 6–8 biological replicates per group).DQuantification of apoptotic vessels after fractionated 5 × 3 Gy IR from immunofluorescent images of whole MC38 tumor sections (*n* = 6–8 biological replicates per group).EQuantification of apoptosis in TECs from B16F10 tumor sections (*n* = 4–6 biological replicates per group).FQuantification of apoptotic vessels according to size on thin whole MC38 tumor sections (Ctrl *n* = 4 and treatment *n* = 7 biological replicates).GQuantification of apoptotic vessels according to size in B16F10 tumor sections (Ctrl *n* = 4 and treatment *n* = 10 biological replicates).H, IArea (H) and diameter (I) of apoptotic tumor vessels vs all tumor vessels from segmented thick 80 μm MC38 tumor sections (*n* = 4 biological replicates).J, KRepresentative immunofluorescence image of control (J) and 15 Gy IR‐treated (K) MC38 and B16F10 tumors stained for proliferation (EdU), EC nuclei (anti‐ERG) and tumor vessels (anti‐CD31).L, MQuantification of proliferating cells (L) and proliferating TECs (M) in MC38 tumors (*n* = 8 biological replicates per group).N, OQuantification of proliferating cells (N) and proliferating TECs (O) in B16F10 tumors (*n* = 4 to 9 biological replicates per group).P, QQuantification of proliferating cells (P) and TECs (Q) in MC38 tumors after fractionated IR (*n* = 5–7 biological replicates per group).R, SQuantification of proliferating cells (R) and TECs (S) in B16F10 tumors after fractionated IR (*n* = 5–7 biological replicates per group).T, UMeasurement of proliferating TECs in (T) MC38 tumors (*n* = 9 biological replicates) and (U) B16F10 tumors (*n* = 5 biological replicates) in small (area < 250 μm^2^) and large (area < 250 μm^2^) tumor blood vessels. Data information: Error bars represent mean ± SD, except in (T, U) where box and whiskers plots with min‐max are shown with median being the middle line, *P < 0.05, **P < 0.01, ***P < 0.0001 by analysis of variance (ANOVA), except in (T, U) where *P < 0.05 by paired *t*‐test. Scalebar: 1 mm in A and B (first column) and 100 μm in A and B (all panels except first column) and in (J and K).

We then asked whether the death of TECs resulted from irradiation. In both the MC38 and the B16F10 models, cell death as measured by immunohistochemical staining or flow cytometry (Appendix Fig S1A) for cleaved caspase‐3, co‐localized with CD31, peaked between 48 and 72 h after 15 Gy IR (Fig 4A–C and Appendix Fig S1B–Bl and C) and 24 and 48 h after the last fraction in the fractionated setting (MC38 only, Fig 4D and Appendix Fig S1Bii). However, the extent of cell death differed between the models with substantially more TEC cell death noted in the MC38 (Fig 4C) than the B16F10 model (Fig 4E). This timing is in accordance with reports from others (Moding *et al*, 2015). Tumor cell death also occurred over approximately the same time course after single and fractionated IR (Appendix Fig S1Biii and Biv).

**Figure EV5:**
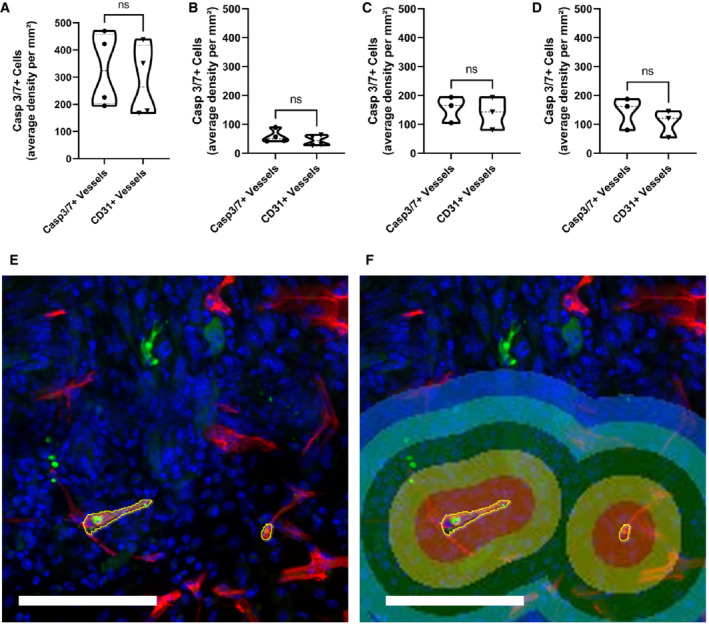
Average density of Caspase‐3/7‐positive cells close to blood vessels A–F The density per mm^2^ of Caspase‐3/7 (Casp 3/7)‐positive cells within 100 μm radius from (Casp 3/7)‐positive TECs was determined at 48 h after single‐dose irradiation to (A) MC38 tumors, (B) B16F10 tumors or (C) 48 h and (D) 168 h after the first dose of 5x 3 Gy of fractionated irradiation in MC38 tumors. A representative image of (E) MC38 tumor vasculature with Casp 3/7‐positive TEC and other cells with the (F) overlaid analysis mask. Tumor sections were stained with anti‐CD31 (red), anti‐Cas 3/7 (green) and counterstained with Hoechst (blue). Scale bar: 100 μm. Data information: Presented are violin plots of all data with dashed line representing median and dotted lines quartiles. *n* = 3–4 biological replicates per group. ns—*P* > 0.05 by one‐way analysis of variance with multiple comparisons (ANOVA).

To ask how extensive TEC cell death might be compatible with retention of vascular function, we divided tumor vessels into large, medium, and small based on surface area and partitioned these compartments for analysis. Most of the cleaved caspase‐3‐positive TECs were found in the small vessels, not the medium or large vessels (Figs 4F and G). Further, after IR, the numbers of cleaved caspase‐3 endothelial cells only increased in the small vessels. As an alternative approach, we stained thick 60–80 μm sections at 48 and 72 h after IR for cleaved caspase‐3 (Fig 4B). The majority of cleaved caspase‐3‐positive TECs were in sprouts and blunt‐ended vessels (Fig 4B). Segmentation of tumor blood vessels of thick sections and respective measurement of vessel diameter, area, and length confirmed that most of the apoptotic endothelial cells were in small vessels and sprouts (Fig 4H and I, and Appendix Fig S1E). Thus, TEC cell death after irradiation was less prominent in the large vessels that were also the perfused vessels. Moreover, the death of TECs was not associated with increased apoptosis of adjacent tumor cells, as the average density of caspase‐3‐positive cells was not significantly increased within 100 μm of the caspase‐3‐positive TEC compared with the viable vasculature (Fig EV5A–F).

IR leads to DNA damage, which can lead to apoptosis but alternatively can result in cell cycle arrest. We asked whether TEC proliferation was affected by irradiation by injecting mice bearing tumors with the proliferation marker EdU 2 h before tumor harvest (Fig 4J and K). Flow cytometry and immuno‐staining for EdU confirmed that both single and fractionated IR reduced overall proliferation in MC38 (Fig 4L and P) and B16F10 tumors (Fig 4N and R). TEC proliferation was comprehensively blocked and reduced as early as 12 h after single‐dose IR in MC38 tumors (Fig 4M and Appendix Fig S1C–Cii) and TECs showed little capacity to recover and resume the cell cycle after this dose of IR (Fig 4M and Appendix Fig S1C–Cii). Fractionated irradiation elicited a similar response from TECs (Fig 4Q). In contrast, tumor cells showed reduced, but continuing proliferative capacity after IR (Appendix Fig S1D–Dii). TECs from B16F10 tumors (Fig 4J, K, O, and S) showed a similar pattern with a prolonged inhibition of TEC proliferation (Fig 4F). Interestingly, when examining EdU‐positive TECs in control non‐irradiated tumors, we observed that the smaller vessels are more proliferative than large vessels in both tumor models (Fig 4T and U), which could also explain the more prominent apoptosis observed in small vessels after IR (Fig 4H and I).

### Gene expression of irradiated tumor endothelial cells

**Figure 5:**
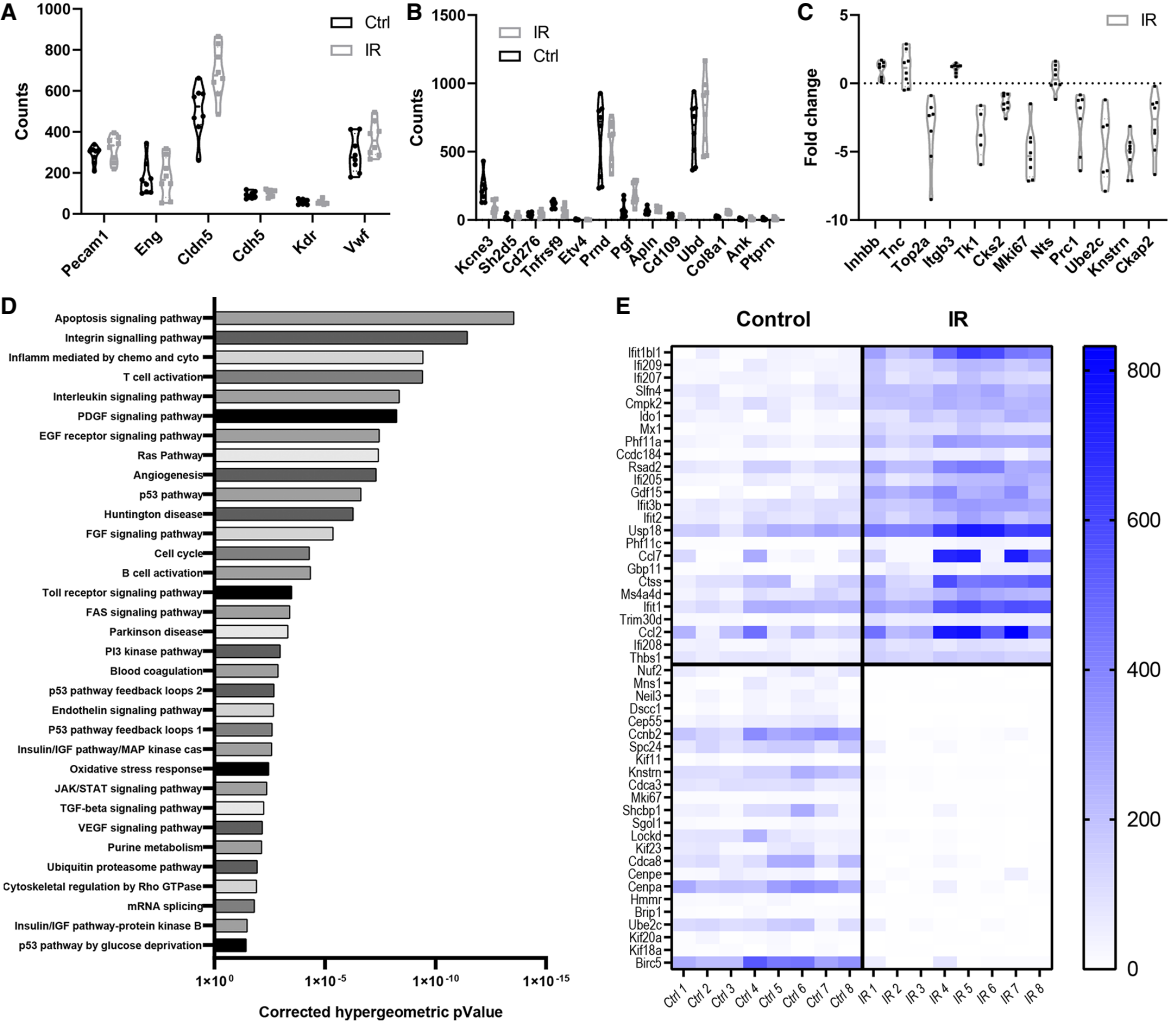
Differential expression analysis in bulk RNAseq data from FACS‐sorted TECs isolated from MC38 tumors 48 h after 15 Gy single‐dose IR AViolin plot of expression of common EC markers present in TECs from MC38 tumors.BViolin plot of expression of previously published TEC marker genes in TECs from MC38 tumors.CDownregulation of angiogenesis genes in TECs from MC38 tumors. Dotted line represents no change in expression in comparison with Ctrl.DPanther classification pathway analysis of the most predominant pathways upregulated in TECs from MC38 tumors.ETop 25 up (top half) and down‐regulated (bottom half) genes in the dataset from TECs from MC38 tumors. Data information: Ctrl—TEC from non‐irradiated MC38 tumors. IR—TEC from irradiated MC38 tumors. *n* = 8 biological replicates per group. Color scale in (E) represents gene counts.

We then characterized gene expression levels in bulk FACS‐sorted TECs isolated from MC38 tumors 48 h after 15 Gy single‐dose irradiation (Appendix Fig S2). Evaluation of expression of well‐recognized EC gene sets or of tumor‐associated EC markers showed that their gene expression was consistent with their TEC origin (Fig 5A and B). The expression of these genes did not show significant changes after radiation. There was a consistent reduction in the expression of genes associated with angiogenesis after irradiation (Fig 5C). No differences were seen in the expression of genes specifically associated with tip, stalk, or phalanx cells (Appendix Fig S3A–C). Notably, Panther pathway analysis of gene expression revealed upregulation of apoptotic pathways (Fig 5D). Additionally, p53 pathways which can affect both apoptosis and cell cycle checkpoints were also upregulated after radiation in TECs (Fig 5D). Evaluation of the differences in the affected pathways with MetaCore revealed further changes in cell cycle regulation including that of the DNA damage‐activated pathways directed by ATM/ATR (Appendix Fig S3D). There was further suggestion of activation of interferon‐based pathways, which would also be consistent with DNA damage, as majority of the most differentially expressed genes were from these pathways (Fig 5E, Appendix Fig S3D).

**Figure 6:**
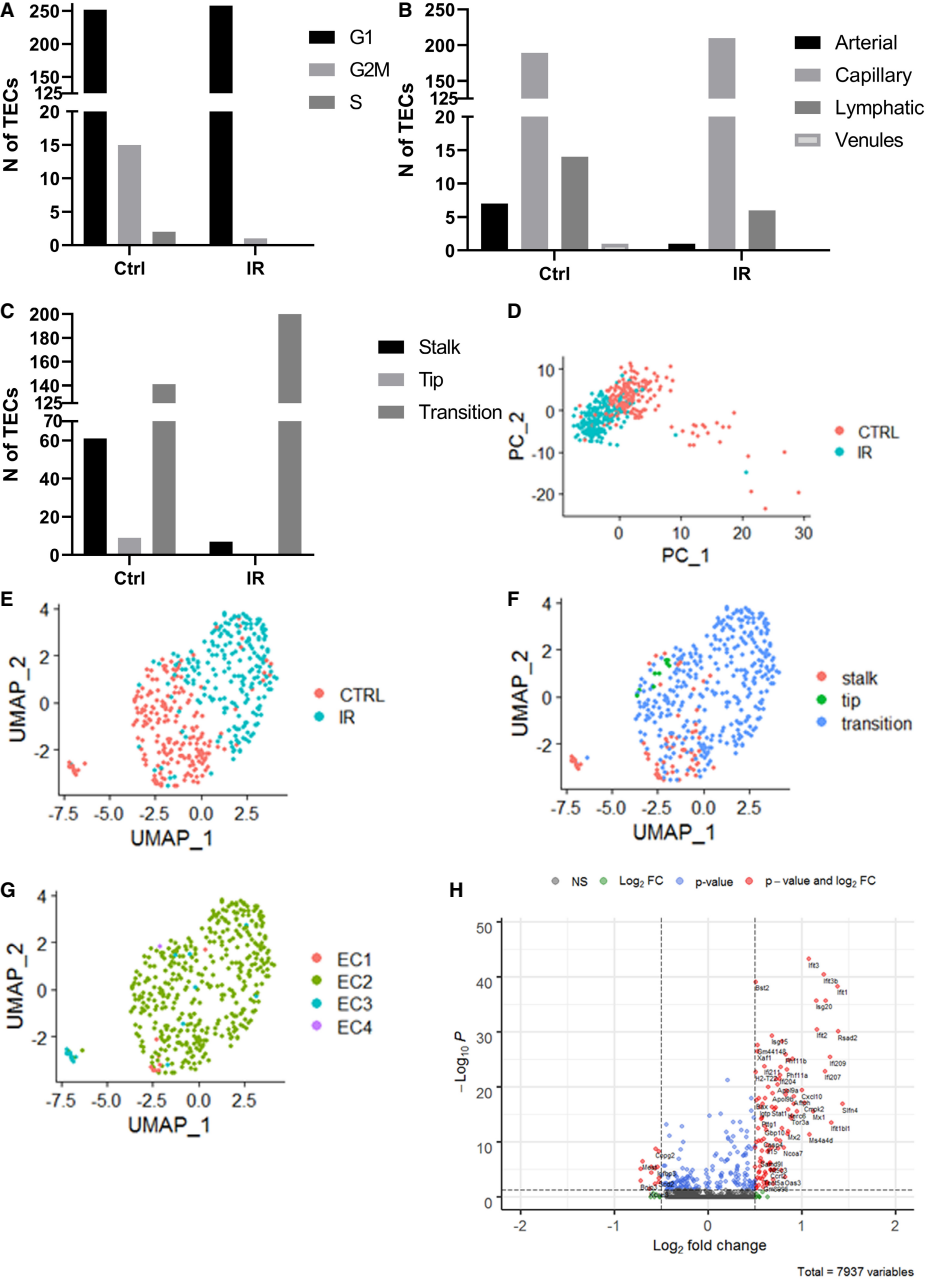
Single‐cell RNA sequencing of TECs 48 h after 15 Gy single‐dose IR ANumber of TECs in different cell cycle phases. The cyclone function from the scran package version 1.14.6 (Lun *et al*, 2016) was used to assign cell cycle phase to cells.BNumber of TECs with an arterial, capillary, lymphatic, or venular gene signature. Label transfer from Zhao *et al* (2018a) was based on the *FindTransferAnchors* (*dims = 1:30*) and *TransferData* (*dims = 1:30*) functions. Both reference and query set were processed with *NormalizeData* and *FindVariableFeatures* (as shown above). Significance between specific labels in control and irradiated cells was determined by repeating the labeling in 100 random samples with replacements (bootstrap) and testing for differences with the Wilcox rank‐sum test.CNumber of TECs with gene signatures specific for stalk, tip, or transition subtypes. Label transfer from Zhao *et al* (2018a) was based on the *FindTransferAnchors* (*dims = 1:30*) and *TransferData* (*dims = 1:30*) functions. Both reference and query set were processed with *NormalizeData* and *FindVariableFeatures* (as shown above). Significance between specific labels in control and irradiated cells was determined by repeating the labeling in 100 random samples with replacements (bootstrap) and testing for differences with the Wilcox rank‐sum test.DPrincipal component analysis plot of TECs per condition.EClustering of TECs according to their gene expression.FOverlay of stalk, tip, and transition TEC subtypes on the clustering generated in (E).GOverlay of EC1: Arterial, EC2: Capillary, EC3: Lymphatic, EC4: Venules subtypes on the clustering generated in (E).HDifferentially expressed genes in the capillary cells. Dashed lines represent threshold above which the genes were considered as differentially expressed. Data information: Ctrl—TEC from non‐irradiated tumors. IR—TEC from irradiated tumors.

To further explain which TEC subtypes are responsible for the observed changes in gene expression, we performed single‐cell RNA sequencing in FACS‐sorted TECs isolated from the same MC38 tumors as for bulk sequencing 48 h after 15 Gy single‐dose irradiation (Appendix Fig S2). We observed an almost complete annihilation of TEC in G2M and S phase of the cell cycle after IR (Fig 6A) confirming our EdU and IF staining observation. The G2M and S phase TECs were then excluded from further analysis to avoid having a strong cell cycle effect in our data. Next, we used the EC subtype and stalk/tip‐like subtype EC labels from Zhao *et al* (2018a), Data ref: Zhao *et al* (2018b) to cluster our data and showed that majority of the TECs in our dataset have the capillary subtype signature (Fig 6B) and transition cell signature (Fig 6C). There was also a marked decrease in arterial (Fig 6B) as well as stalk‐type TECs after IR (Fig 6C). Further, principal component analysis showed that PC2 (and to some extend PC1) is correlated with the treatment (Fig 6D). We have then performed unsupervised clustering, which revealed that irradiated and control cells form separate clusters (Fig 6E) and that control cells are a more heterogeneous population in terms of EC subtypes and stalk/tip‐like subtypes (Fig 6F and G). Due to the number of cells in each subtype, we could only look at the differentially expressed genes in the capillary‐like cells which confirmed the bulk RNAseq data on the activation of interferon‐based pathways (Fig 6H).

## Discussion

Here, we show that radiation induces endothelial cell death in tumors, yet the effects on vascular structure are minimized because the death occurs preferentially in small non‐perfused vessels. On the one hand, vascular injury has been proposed as a prominent factor governing tumor response to radiation therapy. Single doses of 12 Gy to murine tumors were reported to lead to apoptosis of the endothelial cells. On the other hand, vascular function was often minimally perturbed at these doses (Garcia‐Barros *et al*, 2003; Kim *et al*, 2015). Moreover, in patients undergoing radiation therapy for cancer, reoxygenation often results (Hong *et al*, 2016), suggesting that vascular structure might be conserved in some cases.

Further, the Kirsch's laboratory has elegantly shown through genetic means that tumor cell sensitivity to radiation is a critical factor in tumor response while impairment of apoptosis in the tumor vasculature did not necessarily alter tumor growth (Moding *et al*, 2015; Torok *et al*, 2019). We undertook this study to observe the vascular endothelial network in murine tumors with 2‐photon microscopy supplemented with histological observation, gene expression analysis, and DCE‐MRI. Irradiation of tumors with a single dose (15 Gy), previously reported to be lethal to tumor endothelium, led to significant TEC cell death and apoptosis within the tumor vasculature consistent with previous reports. Nonetheless, this was not sufficient to substantially disrupt vascular structure in the irradiated tumors. Fractionated delivery of radiation also had little deleterious effect on the structure of vascular networks. This was the case despite the death of many endothelial cells. Induction of TEC cell death was minimal in the larger vessels that form the bulk of the functional vascular networks and was predominantly confined to the endothelium of small vessels that make little contribution to vascular function. Thus, after irradiation, functional vascular structures remained intact or even improved despite endothelial cell death. Interestingly, we did not observe an increase in the permeability of tumor blood vessels for Qdots 705 after irradiation in our imaging experiments (Fig 3 and Appendix Fig S3). There are contrary reports of increased permeability of tumor blood vessels after irradiation (Moding *et al*, 2013). As our tumor models and contrast agent used, as well as the dose of irradiation that was delivered, differ from the ones in Moding *et al*, the results are not directly comparable. The measured change in the permeability of tumor vasculature depends on the tumor model, the size and type of particles used to determine the permeability and also on their shape. Thus, direct comparisons between studies are only possible when at least the same particles are used for determining the permeability of tumor vasculature. This is also one of the drawbacks of this study, as we have intentionally opted for Qdots (Qtracker^®^ non‐targeted quantum dots, Thermo Fischer Scientific) that do not extravasate readily and have a long plasma half‐life in comparison with fluorescently labeled dextrans. This choice enabled us to perform daily imaging of the same tumor; however, as a result, we lost the ability to directly compare the changes in the permeability of tumor blood vessels after irradiation to other published studies.

By using vascular labeling based upon genetic marking of endothelial cells, we have been able to visualize the vast majority of endothelial cells within a tumor regardless of perfusion. We studied the response to radiation of two different types of allografted murine tumors, MC38 and B16F10. The extent of vessels with smaller diameters, the average lengths between branches, and the numbers of sprouts were roughly similar for allografts derived from each of these cell lines. However, MC38 was a much more vascular tumor with a substantial number of non‐perfused vessels; >40% in MC38 tumors compared to ~30% in B16F10 tumors. Because TEC cell death after radiation was predominantly in the smaller vessels and because each tumor type had different percentages of smaller vessels, the consequence of radiation was different in each tumor type. These differences could be attributed to the density of the small but poorly or non‐perfused vessels. Radiation of MC38 tumors with highly branched vasculature and many smaller vessels with a single dose of 15 Gy led to a decrease in small vessel numbers. Reduction of perfusion was also not evident despite vessel loss, which was confirmed by DCE‐MRI. Fractionated radiation had little effect on the vascular networks or perfusion of MC38 tumors. In contrast, in B16F10 tumors with fewer smaller vessels and greater distances between branches, radiation also removed smaller vessels, but because they were relatively infrequent in these tumors, there was little consequence to the vascular networks either. In the B16F10 with fewer smaller non‐perfused vessels, much less TEC cell death after radiation was also evident, consistent with the observation that TEC cell death after radiation is confined to the smaller non‐functional vessels or sprouts. Further, after radiation there was no change in perfusion. Thus, the nature of vascular structure change correlated with the extent of small non‐perfused vessels in the tumor.

This distinction perhaps would be predicted based on modeling of vascular networks. Our colleagues using these vascular networks in their models have shown that the removal of smaller vessels leading to a greater ratio between the length between branches (l) and vessel diameter (d) (l/d ratio) would be predicted to increase perfusion by altering the proportion of hematocrit splitting, in a fashion perhaps analogous to vascular normalization (Bernabeu *et al*, 2020). This raises the possibility that knowing the extent of the smaller, non‐perfused vessels, perhaps reflected in the l/d ratio, might allow a prediction of the consequence of radiation on the tumor. If a tumor had a large proportion of small TEC cell death susceptible vessels, then radiation, by eliminating them, might be more likely to lead to improved perfusion and/or reoxygenation than radiation of a tumor with few TEC cell death susceptible vessels. Because reoxygenation midcourse in therapy has been identified as a prognostic marker in head and neck and cervical cancers (Shibuya *et al*, 2011; Lock *et al*, 2017), this work raises the possibility that the vascular structure of the tumor might predict the development of reoxygenation.

This model also has some parallels in vascular normalization. As Jain first pointed out, with anti‐VEGF therapy, perfusion and oxygenation paradoxically improve in tumors. This may result similarly from pruning of the smaller, less functional vessels. Normalization also includes pericyte recruitment, also observed in irradiated tumors (Sörensen *et al*, 2009; Martin *et al*, 2019).

Because the induction of TEC cell death is confined mainly to smaller, non‐functional vessels, their death has little consequence on vascular function. Still, the response of the endothelial cells in the remaining vessels is also likely to contribute to the response to radiation by a tumor. Based on our results, we would expect the remaining viable endothelial cells to be in cell cycle arrest. In addition, much work suggests that they undergo senescence coincident with the cell cycle arrest (Choi *et al*, 2018; Venkatesulu *et al*, 2018). However, the senescent cell remains viable and seemingly capable of acting as a vascular channel. Further, senescent cells in general and irradiated endothelium specifically secrete many cytokines that modify their milieu (Moeller *et al*, 2004; Tchkonia *et al*, 2013; Tavora *et al*, 2014).

Overall, our results help explain why extensive TEC cell death at higher doses of radiation does not necessarily translate into extensive vascular impairment. Further, the nature of the alterations in the vascular network may depend upon the density of small non‐perfused vessels containing more endothelial cells susceptible to apoptosis.

## Materials and Methods

### Animals

C57BL/6J female mice were from Charles River. The C57BL/6‐Tg(Cdh5‐cre/ERT2)1Rha transgenic mice were kindly provided by Prof. Sarah de Val (University of Oxford), and permission to use them was obtained from Prof. Ralf Adams (Max Planck Institute for Molecular Biomedicine), B6.Cg‐Gt(ROSA)26Sortm9(CAG‐tdTomato)Hze/J mice were purchased from Jackson Laboratory (Stock Number: 007909). Female Ve‐CreERT2 mice and male GtRosa26 reporter mice were crossed to obtain C57BL/6‐Tg(Cdh5‐cre/ERT2)1Rha‐Gt(ROSA)26Sortm9(CAG‐tdTomato)Hze/J mice (VE‐TOM mice) and were bred in our facility. All animal experiments were conducted in accordance with the United Kingdom Animals (Scientific Procedures) Act 1986 as amended (Amendment Regulations 2012 [SI 2012/3039]), under the authority of a UK Home Office Project License (PPL 30/2922 and PCDCAFDE0), with local ethical approval from the University of Oxford Animal Welfare and Ethical Review Panel. Mice were randomized to control versus treatment groups, and the investigators were not blinded to the experiments. Collection and stopping points were predetermined. Additionally, the experiments were terminated and mice humanely culled if tumors grew up to the size allowed on our animal license or if the implantation of the imaging window failed.

### Tamoxifen treatment

A 100 μl of tamoxifen (Sigma‐Aldrich) dissolved in corn oil and 5% ethanol at 10 mg/ml was administered via gavage daily for 10 days (5 days of gavage, 2 days of pause, and 5 days of gavage) to induce the expression of TdTomato in endothelial cells. Induction efficiency of tdTomato in endothelial cells (ECs) was checked by observing the ear with an inverted Zeiss LSM 880 microscope under epifluorescence excitation/emission for tdTomato. Mice selected for intravital imaging had an induction efficiency >95% (scored by two independent researchers) and were chosen without specific gender selection.

### Cell lines

Murine colon adenocarcinoma cells (MC38) were a gift from Dr. Lee Gorden (Vanderbilt University, Nashville, TN, USA). Melanoma (B16F10) cells were obtained from American Type Culture Collection. Both cell lines were transduced with EGFP using lentiviruses to obtain MC38 GFP and B16F10 GFP cells. Additionally, MC38 cells were separately transduced with lentiviruses to obtain MC38 mCherry cells. Cells were cultured in DMEM (MC38) and RPMI 1640 medium (B16F10), supplemented with 10% (v/v) fetal bovine serum and 1% penicillin–streptomycin. All cells were maintained in a humidified incubator with 5% CO_2_ at 37°C. Cells were used at passage number < 10 and were routinely tested negative for Mycoplasma with MycoAlertTM Mycoplasma Detection Kit (Lonza).

### Subcutaneous tumor model

A 100 μl of cell suspension in 0.9% NaCl (2.5 × 10^5^ cells) was injected subcutaneously on the right flank. Tumor volumes were measured every other day using a digital caliper. Tumor volumes were calculated using the formula: Length × Width × Depth × π/6.

### *In vivo* radiation treatment

Once tumors reached 80–100 mm^3^, mice were randomly assigned to experimental groups. Mice were anesthetized under inhalation with isoflurane and led shielded with only the tumors exposed to radiation. Tumors received either 15 Gy or fractionated 5 × 3 Gy X‐ray radiation treatment (300 kV, dose rate of 2.25 Gy per minute) delivered to tumors using a Gulmay RS320 irradiation system (Gulmay Medical Ltd). Mice whose tumors ulcerated during experimental timeline were excluded from the study.

### *In vivo* radiation treatment of tumors in abdominal window chambers

Mice were anesthetized under inhalation with isoflurane and placed in an imaging‐guided small animal radiation research platform (SARRP) irradiator (Xstrahl Ltd). A cone beam CT image of each mouse was obtained, and the treatment was planned using Muriplan (Xstrahl Ltd) to ensure uniformity of dose across the tumor while sparing the surrounding normal tissue. This was achieved using a coronal arc beam with the isocenter positioned a few millimeters above the glass window with a beam at an angle of 65° to the vertical and the mouse rotated through 360° horizontally. To achieve full coverage of the tumor, a 4 mm × 10 mm field size (defined as the isocenter and the long axis parallel to the mouse) was chosen. The SARRP was used to deliver 15 Gy of X‐rays (220 kVp copper‐filtered beam with HVL of 0.93 mmCu) to the tumor at ~2 Gy per minute; this was given either in a single fraction or five daily fractionations of 3 Gy X‐ray radiation to the tumor. Dosimetry of the irradiator was performed as previously (Hill *et al*, 2017). A visualization of the planned dose distribution is presented in Appendix Fig S4.

### Tumor allograft model and abdominal window imaging

Abdominal window chamber model in mice allowed for intravital imaging of the tumor vasculature. An abdominal window chamber from either titanium or biocompatible plastic was surgically implanted onto shaved and depilated VE‐TOM mice as described previously (Ritsma *et al*, 2013), with the difference, that only the skin was cut, whereas the abdominal wall was left intact. Then, MC38 GFP or B16F10 GFP tumors were induced by injecting 5 μl of 2.5*10^5^ cells in a 50/50 mixture of saline and matrigel (BD Biosciences) into the thin fat layer above the abdominal muscles. Mice were monitored daily. Once tumors in window chambers reached ~ 4 mm diameter, imaging was started. Mice were anesthetized with inhalation anesthesia with isoflurane and kept on a heated stage inside a dark heated chamber with breathing rate monitored. Upon imaging, Qtracker 705 (Invitrogen) vasculature labels in a 1:10 dilution in sterile saline were continuously injected through a tail vein cannulation connected to a syringe and an automated pump (Harvard Instruments) at an injection rate of 75 μl per hour starting with a bolus injection of 12.5 μl.

Tumor vasculature images were acquired with an inverted Zeiss LSM 880 microscope (Carl Zeiss AG). The microscope was connected to a tunable Mai‐Tai laser (Newport Spectra‐Physics). An excitation wavelength of 940 nm was used, and the emitted light was collected through gallium arsenide phosphide (GaAsP) detectors with a bandpass filter of 524–546 nm for GFP and a 562.5–587.5 nm bandpass filter for TdTomato. A multi‐alkali PMT detector with a bandpass filter of 670–760 nm was used to record Qtracker 705 signal. A Zeiss 20x water immersion objective with NA 1.0 was used to acquire Z stack‐TileScan images with a dimension of 512 × 512 pixels in x and y. Approximately, 70 planes in z, with a step size of 5 μm, were acquired. Voxel size in the x‐y plane was 0.83 μm × 0.83 μm and 5 μm in z.

### Image post‐processing

Intravital images were post‐processed with Imaris (Bitplane) channel arithmetic's due to bleed‐through of GFP into TdTomato channel and bleed‐through of TdTomato into the Qtracker 705 channel. Imaris was also used for visualizing the acquired images.

### Segmentation and quantification of intravital images

Segmentation of the tumor vasculature from the post‐processed intravital images was performed with a previously published segmentation algorithm using the Advanced Research Computing facility in Oxford (preprint: Bates *et al*, 2017).

### EdU and EF5 labeling

For EdU (5‐ethynyl‐2′‐deoxyuridine) labeling of proliferating cells, 200 μl of EdU (Invitrogen) solution in PBS with a concentration of 2 mg/ml was intraperitoneally injected into mice 2 h prior to tumor resection. Tumors were then processed for flow cytometry. For EF5, mice were injected intraperitoneally with EF5 [2‐(2‐nitro‐1/−/−imidazol‐l‐yl)‐N‐(2,2,3,3 ∼ −pentafluoropropyl)acetamide], a nitroaromatic compound stabilized in the absence of oxygen (Lord *et al*, 1993) a kind gift from Prof. Cameron Koch (University of Pennsylvania, Philadelphia, PA, USA) and EdU (Santa Cruz Biotechnology) 2 h before sacrifice. Tumors were then processed for immunofluorescence staining.

### Flow cytometry profiling of tumor endothelial cells

MC38 GFP, MC38 mCherry, and B16F10 GFP tumor‐bearing mice were sacrificed and tumors harvested. Tumors were cut into small pieces and incubated in Hank's balanced salt solution (Thermo Fisher Scientific) with collagenase 2 (Worthington) (10 mg/ml) and DNase I (2 U/ml) for 45 min with shaking at 37°C. Cells were then strained through 50 μm strainers, centrifuged and resuspended in FACS buffer (PBS containing 2% FBS), and stained for: CD45, TER119, CD150, CD31, and LIVE/DEAD (detailed list of antibodies is in Appendix Table S1 and gating strategy in Appendix Fig S2). Proliferation was analyzed with the Click‐iT^®^ EdU Alexa 488 Flow Cytometry Assay Kit (Thermo Fisher Scientific) and apoptosis was analyzed with CellEvent™ Caspase‐3/7 Green Flow Cytometry Assay Kit (Invitrogen), all according to the manufacturer's instructions. Samples were measured using an Attune NxT Flow Cytometer (Thermo Fisher Scientific). Cells were identified based on forward and side scatter, after exclusion of doublets. Tumor endothelial cells were gated as DEAD negative, CD45^−^, CD150^−^, Ter150^−^, GFP^−^ (or mCherry^−^), CD31^+^ positive cells. Cells' relative frequency of each subpopulation from live‐cell gate or an absolute number of each subset were determined. Data were analyzed using FlowJo software (Tree Star Inc.).

#### Dynamic contrast‐enhanced magnetic resonance imaging (DCE‐MRI)

MRI was performed with a 7.0T 210 mm horizontal bore VNMRS preclinical imaging system equipped with 120 mm bore gradient insert (Varian Inc) and a 32 mm ID quadrature birdcage coil (Rapid Biomedical GmbH). DCE‐MRI was performed using a respiration‐gated 3D spoiled gradient echo scan with TR 1.7 ms, TE 0.632 ms, FOV 64 × 32 × 32 mm^3^, matrix 128 × 64 × 64, gradient spoiling with 159 mT/m for 0.432 ms in all three axes, RF hard pulse duration 16 μs, FA 5°, and RF spoiling. Data were acquired in blocks of 64 k‐space lines, and the two data blocks acquired prior to detection of each breath were reacquired immediately after the same breath to give a full 3D scan in 12–15 s. Fifty repeats of the 3D scan were performed with 30 μl of a Gd‐contrast agent (Omniscan, GE Healthcare) infused via a tail vein cannula over 5 s starting at the beginning of frame 11/50. A timestamp corresponding to the center of k‐space was recorded for each frame (Kinchesh *et al*, 2018). Mice were under isoflurane inhalation anesthesia 30% O_2_ 70% air mixture with respiration maintained at 40–60 breaths per minute. Animals were kept warm for MRI using an MR‐compatible electrical rectal probe‐driven heating system (Kersemans *et al*, 2019).

#### T1‐Mapping

A respiration‐gated 3D variable flip angle (VFA) scan (Christensen *et al*, 1974) was performed with 16 flip angles (FAs) ranging from 1° to 8°, and other parameters as for DCE‐MRI, in a scan time of approximately 4 min to enable estimation of T1. A respiration‐gated 3D actual flip angle imaging (AFI) scan was performed to enable a voxel‐wise correction of the FAs prescribed in the VFA scan during T1 analysis (Yarnykh, 2007). AFI scan parameters were TR1 10 ms, TR2 100 ms, TE 0.46 ms, RF hard pulse 128 μs, FA 64°, FOV 64 × 32 × 32 mm^3^, matrix 64 × 32 × 32 and scan time approximately 4 min.

#### DCE‐MRI image segmentation and analysis

A respiration‐gated 3D balanced SSFP (bSSFP) scan with TR 2.8 ms, TE 1.4 ms, FOV 64 × 32 × 32 mm^3^, matrix 256 × 64 × 64, and FA 30° was acquired as an anatomical reference (Gomes *et al*, 2019). bSSFP banding artifacts were minimized with the combination of four phase‐cycled images acquired in approximately 2 min in total using an elliptical signal model (Xiang & Hoff, 2014). Tumors were manually segmented from the obtained bSSFP anatomical reference scan using ITK‐Snap (Yushkevich *et al*, 2006). Conversion from MRI signal to Gd concentration was done according to the description given in Schabel *et al* (Schabel & Parker, 2008). Mean residence time (MRT) (Yamaoka *et al*, 1978) and initial area under the curve for the first 90 s post‐injection (iAUC90) (Robinson *et al*, 2003) were calculated at each voxel location within the tumor. As perfused tumor voxels the tumor voxel whose iAUC90 value was greater than the median value for muscle were counted (Robinson *et al*, 2003). All processing was done using in‐house software written in Matlab (The Mathworks).

### Immunofluorescence staining

Mice were euthanized, and tumors were resected and immediately placed in 4% PFA in PBS and incubated overnight at 4°C. Samples were then placed into 30% sucrose in PBS solution (w/v) overnight. Tumors were embedded in optimal cutting temperature (OCT) medium, frozen in liquid nitrogen and stored at −80°C. Thin 10 μm or 60 μm cryosections were cut with a Leica CM1950 (Leica Biosystems) cryostat on glass slides (VWR). Tumor sections were air‐dried, washed with PBS, and blocked with 5% BSA/5% donkey serum (v/w) in PBS containing 0.25% Triton X‐100 for 1 h at room temperature in a humidified chamber. Sections were stained for proliferation with the Click‐iT EdU (5‐ethynyl‐2′‐deoxyuridine) Alexa647 immunofluorescence staining kit (Invitrogen) according to the manufacturer's instruction followed by incubation with primary antibodies in blocking solution overnight at 4°C. The following primary antibodies were used: CD31, ERG, CD45, GR1, CD68, and cleaved caspase‐3 (detailed list of antibodies in Table S1). Sections were washed three times in PBS and incubated with Alexa Fluor 488‐, Alexa Fluor 546‐, or Alexa Fluor 647‐conjugated secondary antibodies (Invitrogen, 1:500) for 1 h at room temperature in a humidified chamber. Sections were washed three times in PBS and counterstained with Hoechst 33342 (Sigma‐Aldrich) and washed in PBS. Sections were mounted with ProLong Diamond Antifade Mountant (Molecular Probes). Whole tumor images were acquired with a brightfield (Nikon Ni‐E) or inverted confocal microscope (Andor Dragonfly, Oxford Instruments) and processed using Imaris (Bitplane) or HALO (Indica Labs) image analysis software for spatial analysis.

For thick 100–200 μm sections, tumors were resected and fixed in 4% PFA in PBS overnight and placed in 0.25% low melting agarose in PBS solution and cut using a Vibratome (Campden Instruments). Subsequent processing of tissues was the same as for thin section.

### RNA sequencing: tumor stromal cell preparation

Dissected tumors were finely chopped with scalpels and the obtained fragments digested in HBSS (with Calcium and Magnesium; GIBCO) containing a mix of Collagenases 1 and 3 (Worthington; 3 mg/ml), dispase II (Roche; 7 mg/ml), DNase I (Invitrogen; 2 U/ml) and left at 37°C with gentle agitation for 20 min. The dissociated cells were centrifuged at 500 *g* for 5 min, resuspended in PBS‐5% FBS, and filtered through 50 μm cell strainers before being counted and used for flow cytometry.

### RNA sequencing: flow cytometry of tumor endothelial cells

A total of 25 × 10^6^ cells were stained in a final volume of 100 μl of antibody mix for 15–20 min, in ice, protected from light. Cells were subsequently washed with 2 ml PBS‐5% FBS, centrifuged at 500 *g* for 5 min, and resuspended in 500 μl PBS‐5% FBS. 7‐AAD was added right before the sample acquisition. Sorting was performed by using a BD FACSAria™ Fusion Cell Sorter (BD Biosciences). The antibodies used are listed in Table S1. One hundred cells per population were sorted in 4 μl of lysis mix (0.4% Triton X + RNAse Inhibitor (1:20), dNTPS (10 mM), Oligo dT (10 μM)—according to the original Smart‐seq2 protocol (Picelli *et al*, 2014)) for bulk RNAseq, and 1 cell per well of a 96‐well plate for single‐cell RNAseq, and immediately processed for RNA sequencing or stored at −80°C.

### RNA sequencing: cDNA libraries using Smart‐seq2 protocol

5.7 μl of retro‐transcription mix (see reference for details) was added to each sample. Retro‐transcription was carried out according to the original Smart‐seq2 protocol and cDNA was then pre‐amplified for 15 cycles (Picelli *et al*, 2014). After PCR pre‐amplification, cDNA was purified using Ampure XP magnetic beads according to the manufacturer's instructions, in a ratio of 0.8 to 1 with cDNA, resuspended in 17.5 μl of buffer EB (Qiagen) and stored at −20°C. Quality and concentration of the cDNA generated were assessed using High‐Sensitivity Bioanalyzer kit (Agilent).

### RNA sequencing: Illumina library preparation and sequencing

1 ng of pre‐amplified cDNA was tagmented and indexed with Nextera XT DNA Sample Preparation kit (Illumina) according to the manufacturer's instructions. The product was purified with AMPure XP beads (1:1 ratio) and eluted in 17.5 μl of EB buffer (Qiagen). Samples were loaded on a High‐Sensitivity DNA chip (Agilent Technologies) to check for library size and quality and concentration measured with Qubit High‐Sensitivity DNA kit (Invitrogen). Libraries were pooled to a final concentration ranging between 2 nM and 10 nM and sequenced with either Illumina NextSeq 550 at the MRC Weatherall Institute of Molecular Medicine or Illumina Hiseq 4,000 (25 bp single‐end read) at the Wellcome Trust Centre for Human Genetics in Oxford.

### Analysis of bulk RNAseq data

After the adapters for sequencing were trimmed from single‐end reads in samples (*n* = 8 per group), we aligned the reads to the mouse reference genome GRCm38/mm10 along with transcriptome information by Bowtie 2.2.6 and tophat2 v2.1.0. The aligned reads were used for estimating fold change of normalized expression level, FPKM (Fragments Per Kilobase of transcript per Million mapped reads), for each gene by cufflinks‐2.2.1. The consistency of significant change, false‐positive rate < 0.05, was estimated using the package of non‐parametric rank product in R with the contrast between normal and irradiated cells. The differentially expressed gene set was put into GeneCodis web‐based tool (http://genecodis.genyo.es/) to perform Panther pathways enrichment analysis (Carmona‐Saez *et al*, 2007). Additionally, the differentially expressed gene set was also analyzed with pathway analysis package GeneGo MetaCore (https://portal.genego.com/) to build biological networks and list the associated biological processes and diseases. A *P*‐value of 0.05 was used as a cutoff to determine significant enrichment of a pathway or annotated gene groups present in the MetaCore database.

### Analysis of single‐cell RNAseq data

After the adapters were trimmed out, the reads were aligned to the mouse reference genome GRCm38/mm10 using transcriptome information GRCm38 release 88 by Bowtie 2.2.6 and tophat2 v2.1.0. Reads having highest alignment scores were kept if being aligned to several locations. The number of reads aligned to each gene was counted by HTSeq v0.9.1. All subsequent analysis was performed using RStudio version 1.2.5033 with R version 3.6.3.

Cell QC involved the following criteria (Luecken & Theis, 2019; Amezquita *et al*, 2020): total number of counts per cell, total number of features per cell, and percentage of mitochondrial DNA. Thresholds for the first two parameters were determined by using the median value and median absolute deviation (MAD) as guidance. Cells within 3 MADs of the median value were kept for downstream analysis, that is, total counts > 139,691 and total counts < 887,845, total features > 2,673, and total features < 9,384. Cells with a percentage for mitochondrial genes <5% were considered in the downstream analysis. Furthermore, the *cyclone* function from the scran package version 1.14.6 (Lun *et al*, 2016) was used to assign cell cycle phase to cells. A total of 18 cells labeled as G2M or S phase (the other 510 cells as G1 phase) were removed to avoid cell cycle effects in the downstream analysis. The total number of cells passing cell QC was 428 out of 528 cells. Finally, genes that were expressed in >5 cells were kept for downstream analysis.

The data were processed with the Seurat package version 3.1.4. (Stuart *et al*, 2019). Normalization was based on the *NormalizeData* function (*normalization.method = “LogNormalize*,*” scale.factor = 10*,*000*). Highly variable genes were determined with the *FindVariableFeatures* function (*selection.method = “vst*,*” nfeatures = 2000*). The data were subsequently scaled with the *ScaleData* function.

Principal component analysis was done with the *RunPCA* function using the highly variable genes. Clustering was based on the first 10 principal components; the *FindNeighbors* and *FindClusters* (*resolution = 0.4*) functions were used.

Label transfer from Zhao *et al* (2018a), Data ref: Zhao *et al* (2018b) was based on the *FindTransferAnchors* (*dims = 1:30*) and *TransferData* (*dims = 1:30*) functions. Both reference and query set were processed with *NormalizeData* and *FindVariableFeatures* (as shown above). Significance between specific labels in control and irradiated cells was determined by repeating the labeling in 100 random samples with replacements (bootstrap) and testing for differences with the Wilcox rank‐sum test.

Finally, differential expression analysis between control and irradiated cells was performed only on cells recognized as capillary as this was the only EC subtype with sufficient number of cells in both classes. The Wilcox rank‐sum test was used for differential expression analysis, and *P*‐values were adjusted with the Bonferroni correction.

### Statistics

All values in this study represent means (M) and standard deviations (SD) with the exception that bars in the comparison of the vasculature parameters show means and the standard errors of the mean (SEM). All statistical analyses were performed in GraphPad Prism v8.0. *P* < 0.05 was considered significant. No statistical method was used to predetermine sample size, and experiments were not randomized. The investigators were not blinded to allocation during experiments and outcome assessment. Throughout the manuscript, the following symbols for statistical significance are used: *P < 0.05, **P < 0.01, and ***P < 0.001.

## Author contributions

**Jakob R Kaeppler:** Conceptualization; data curation; formal analysis; investigation; visualization; methodology; writing – original draft; writing – review and editing. **Jianzhou Chen:** Data curation; investigation; methodology; writing – original draft; writing – review and editing. **Mario Buono:** Data curation; formal analysis; investigation; visualization; writing – original draft; writing – review and editing. **Jenny Vermeer:** Investigation; methodology; writing – original draft; writing – review and editing. **Pavitra Kannan:** Investigation; methodology; writing – original draft; writing – review and editing. **Wei‐Chen Cheng:** Data curation; formal analysis; investigation; visualization; methodology; writing – original draft. **Dimitris Voukantsis:** Data curation; formal analysis; investigation; visualization; methodology; writing – original draft. **James M Thompson:** Investigation; visualization; methodology; writing – original draft; writing – review and editing. **Mark A Hill:** Supervision; methodology; writing – original draft; writing – review and editing. **Danny Allen:** Data curation; formal analysis; visualization; methodology; writing – original draft. **Ana Gomes:** Formal analysis; investigation; visualization; methodology; writing – original draft. **Veerle Kersemans:** Formal analysis; investigation; visualization; methodology; writing – original draft. **Paul Kinchesh:** Data curation; investigation; methodology; writing – original draft. **Sean Smart:** Resources; supervision; validation; visualization; methodology; writing – original draft; project administration. **Francesca Buffa:** Supervision; validation; visualization; methodology; writing – original draft. **Claus Nerlov:** Resources; data curation; formal analysis; supervision; validation; writing – original draft; project administration; writing – review and editing. **Ruth J Muschel:** Conceptualization; supervision; funding acquisition; validation; visualization; writing – original draft; project administration; writing – review and editing. **Bostjan Markelc:** Conceptualization; resources; data curation; formal analysis; supervision; funding acquisition; validation; investigation; visualization; methodology; writing – original draft; project administration; writing – review and editing.

## Disclosure and competing interests statement

The authors declare that they have no conflict of interest.

## Supporting information




Appendix S1

Expanded View Figures PDF

## Data Availability

The datasets analyzed during this study are available from the corresponding authors on request. The RNAseq data discussed in this publication have been deposited in NCBI's GeneExpression Omnibus and are accessible through GEO Series accession number GSE168481 (https://www.ncbi.nlm.nih.gov/geo/query/acc.cgi?acc=GSE168481). The uncropped images on which Figs 2 and 3 are based have been deposited in the BioStudies database and are accessible through accession number S‐BSST747 (https://www.ebi.ac.uk/biostudies/studies/S‐BSST747).
