# Supplementary material for: Endothelial cell death after ionizing radiation does not impair vascular structure in mouse tumor models
Source: EMBO Rep. 2022 Jul 18;23(9):e53221. doi: 10.15252/embr.202153221 (PMC9442312; doi:10.15252/embr.202153221)
Supplement: Supplementary file 1 — Appendix S1 [file EMBR-23-e53221-s002.pdf]

Appendix:

Table of Content

1. **Appendix Figure S1:** Flow cytometry analysis of TEC apoptosis and proliferation after single and fractionated IR ………………………………………………………...Page 2
2. **Appendix Figure S2:** TEC FACS analysis …………………………….…………..…Page 3
3. **Appendix Figure S3:** Differential expression analysis of TECs 48 h after 15 Gy single dose IR ……………………………………………………………………………Page 4
4. **Appendix Figure S4:** A visualization of the dose delivered to the tumor with SARRP .. ……………………………………………………………………………….Page 5
5. **Appendix Table S1** ……...…………………………………………………………Page 6

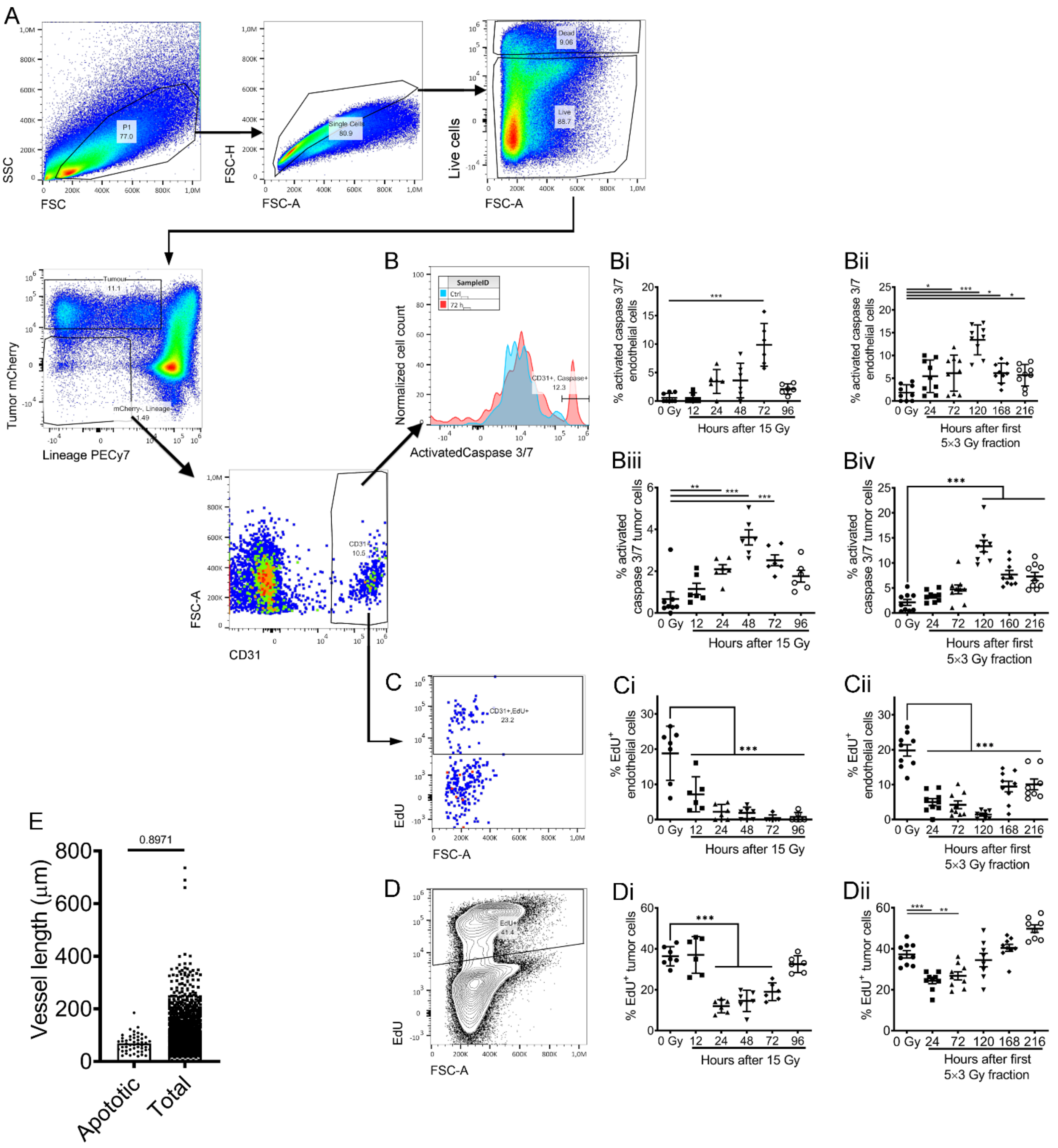

Appendix Figure S1. Flow cytometry analysis of TEC apoptosis and proliferation after single and fractionated IR. (A) representative flow cytometry gating strategy to determine TECs from MC38 tumors. **(B)** histogram of activated caspase 3/7 in TECs and **(Bi)** quantification of activated caspase 3/7 in TECs after single dose IR and **(Bii)** fractionated IR (n=6 biological replicates per group). **(Biii and Biv)** quantification of activated caspase 3/7 in MC38 tumor cell (n=6 biological

replicates per group). **(C - Cii)** histogram and quantification of TECs proliferation after single and fractionated IR in MC38 tumors (n=6-9 biological replicates per group). **(D – Dii)** quantification of proliferating MC38 tumor cells after single and fractionated IR (n=6-9 biological replicates per group). **(E)** vessel length of apoptotic tumor vessels vs all tumor vessels from segmented B16F10 tumor sections. Error bars represent mean ± SD, *P <0.05, **P <0.01, ***P <0.0001 by analysis of variance (ANOVA).

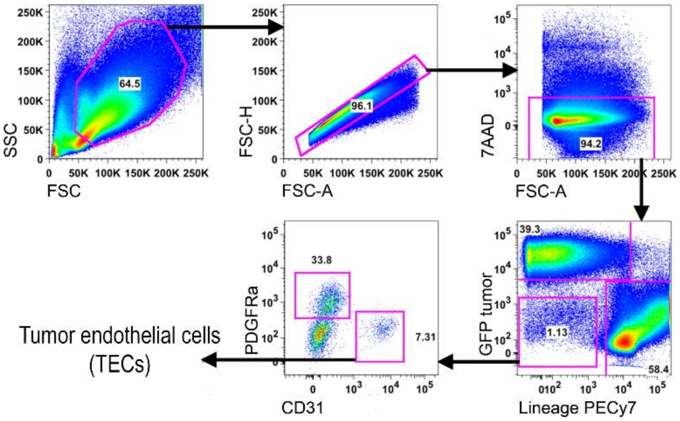

Appendix Figure S2. TEC FACS analysis. Gating strategy used to identify TECs population with the total live (7AAD-) tumor single cell population: TECs:GFP-, Lineage- (CD45-, CD150-, TER119), CD31+, CD144+, and CD105+.

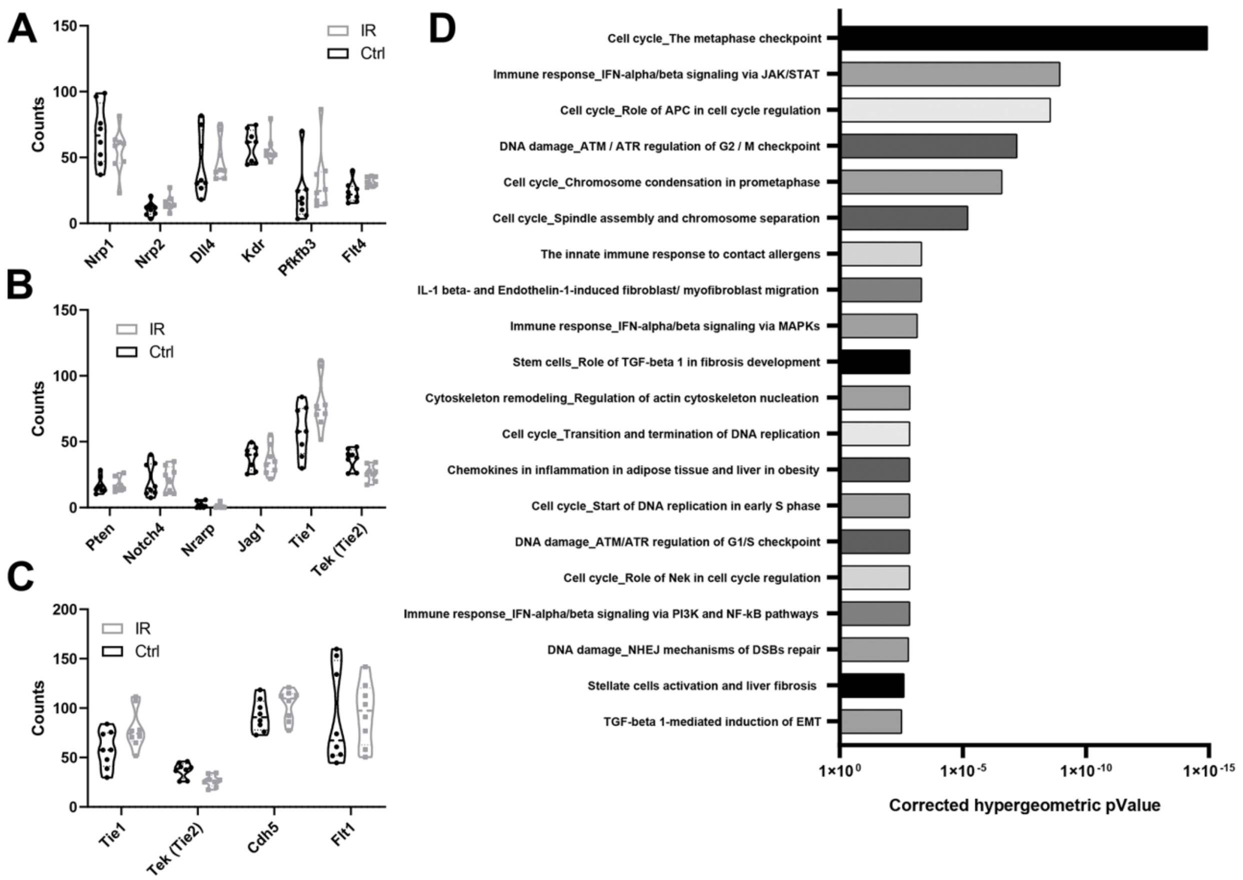

Appendix Figure S3. Differential expression analysis of TECs 48 h after 15 Gy single dose IR. (A) Histogram of marker genes for endothelial tip cells. **(B)** Histogram of marker genes for endothelial stalk cells. **(C)** Histogram of marker genes for endothelial phalanx cells **(D)** Top 20 classified MetaCore signaling pathways in TECs from MC38 tumors. n=8 biological replicates per group.

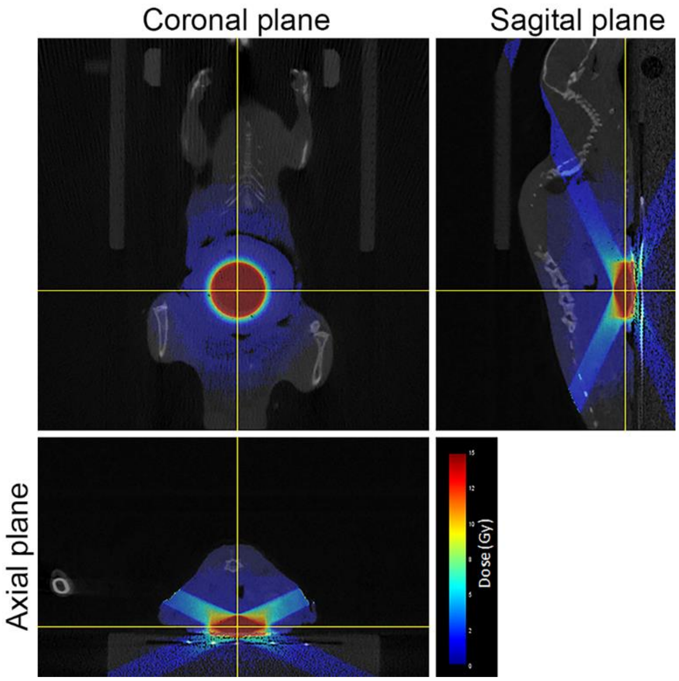

Appendix Figure S4. A visualization of the dose delivered to the tumor with SARRP, using a 4 mm x 10 mm x-ray beam delivered at an angle of 65o to the vertical and the mouse rotated through 360o horizontally. Dose is presented as an overlaid heat-map on Cone Beam CT image with blue color representing 0 Gy and red 15 Gy.

Appendix Table S1

Antibody	Source	Cat#	Dilution	Application
Rabbit anti mouse ERG	Abcam	ab92513	1:200	IF
Rat anti mouse CD45	Abcam	Ab25386	1:200	IF
Rat anti mouse GR1	Abcam	Ab25377	1:200	IF
Rat anti mouse CD68	GeneTex	GTX41865	1:200	IF
Goat anti mouse CD31	R&D	AF3625	1:200	IF
BV421 anti-mouse CD45 (clone 30-F11)	BioLegend	103134	1:600	FC
BV421 anti-mouse TER-119 (clone TER119)	BioLegend	116234	1:100	FC
BV421 anti-mouse CD150 (clone TC15-12F12.2)	BioLegend	115943	1:100	FC
PE/Cy7 anti-mouse CD31 (clone 390)	BioLegend	102418	1:250	FC
Donkey anti-goat Alexa Fluor 647	JacksonImmuno Research	705-605-147	1:400	IF
Donkey anti-goat Alexa Fluor 555	ThermoFisher	A-21432	1:400	IF
Donkey anti-rabbit Alexa Fluor 488	ThermoFisher	A-21206	1:400	IF
Donkey anti-rat DyLight 405	JacksonImmuno Research	712-475-150	1:400	IF
PE/Cy7 anti-mouse CD45 (clone 30-F11)	eBioscience	25-0451-82	1:600	FACS
PE/Cy7 anti-mouse Ter119 (clone TER119)	eBioscience	25-5921-82	1:100	FACS
PE/Cy7 anti-mouse CD150 (clone TC15-12F12.2)	Biolegend	115914	1:100	FACS
PerCPef710 anti-mouse CD31 (clone 390	eBioscience	46-0311-82	1:250	FACS
EF5 compound	University of Pennsylvania by Dr. Cameron Koch	http://www.hypoxia-imaging.org/index.html	10 mM, 250 µg/mouse	IF
anti-EF5 antibody (ELK3-51) Cy3	University of Pennsylvania by Dr. Cameron Koch	http://www.hypoxia-imaging.org/index.html	75 µg/ml	IF

α-EF5 (ELK3-51) Competed Stain Cy3	University of Pennsylvania by Dr. Cameron Koch	http://www.hypoxia-imaging.org/index.html	75 μg/ml	IF
Rabbit anti-mouse NG2 Chondroitin Sulfate Proteoglycan Antibody	MERCK Millipore	AB5320	1:200	IF
