# Supplementary material for: Endothelial cell death after ionizing radiation does not impair vascular structure in mouse tumor models
Source: EMBO Rep. 2022 Jul 18;23(9):e53221. doi: 10.15252/embr.202153221 (PMC9442312; doi:10.15252/embr.202153221)
Supplement: Supplementary file 2 — Expanded View Figures PDF [file EMBR-23-e53221-s001.pdf]

Expanded View Figures

Figure EV1. Evaluation of tdTomato-positive TECs in VE-TOM mice.

A Representative low-power microscopic images (top row) of vasculature from tissue sections of organs and tumors expressing tdTomato (red) in endothelial cells.
B Representative high-power microscopic images of the same tissue section expressing tdTomato.
C, D Samples are stained for CD31 (white, C) and CD68 (green, D).
E, F Merged channels of B + C (E) and B + D (F).
G Representative high-power microscopic image of tdTomato (red) and CD45 (green).
H Representative high-power microscopic image of tdTomato (red) and GR1 (green).
I Flow cytometry, gating strategy, and histogram of MC38 tumor TECs 30 days after tamoxifen administration. Percent of tdTomato-positive CD31-positive TECs (red) and FMO (blue).

Data information: Scale bar in (A); 1 mm; scale bars in (B–H): 50 μm.

 © 2022 The Authors

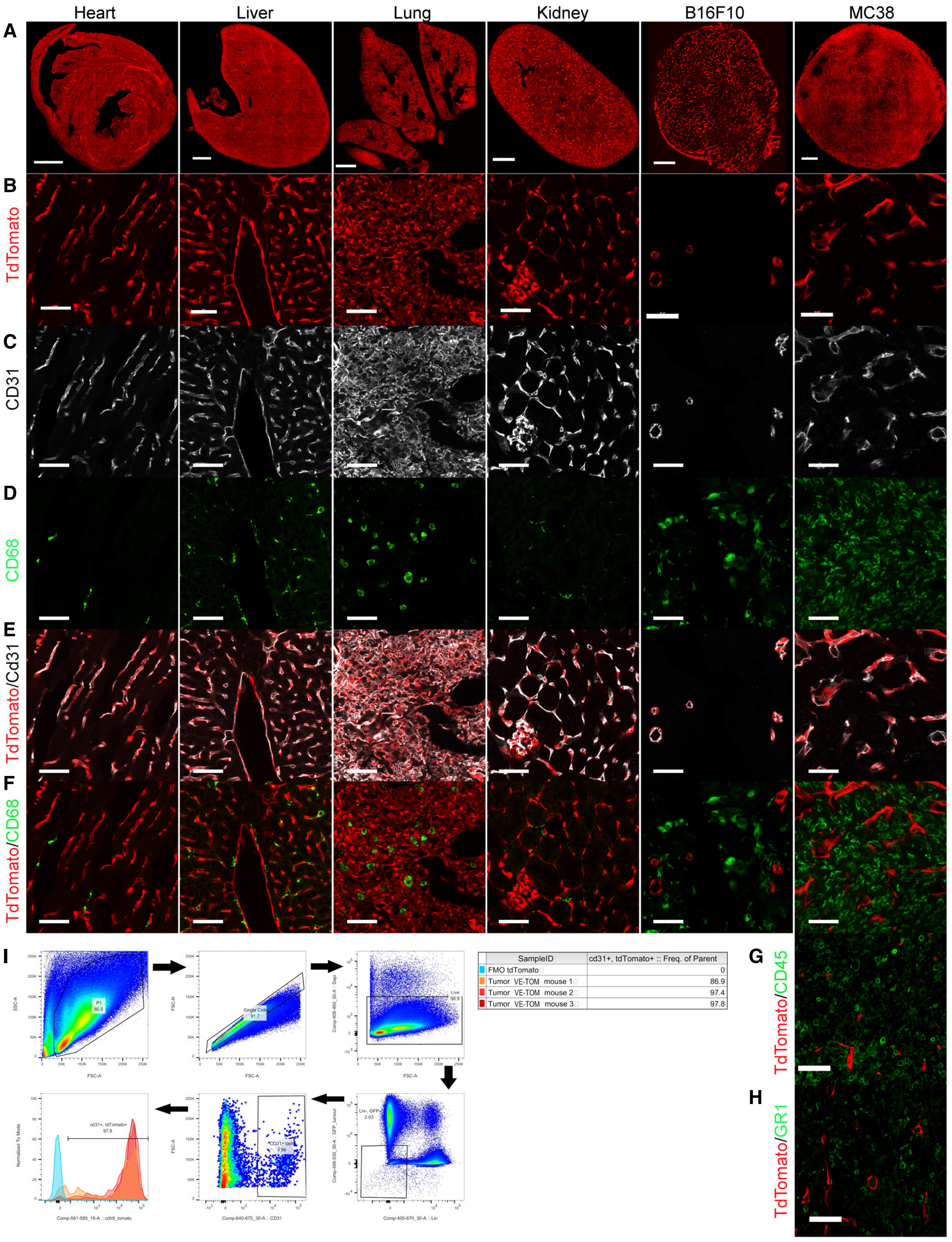
Heart
Liver
Lung
Kidney
B16F10
MC38
A
B
TdTomato
C
CD31
D
CD68
E
TdTomato/Cd31
F
TdTomato/CD68
G
TdTomato/CD45
H
TdTomato/GR1
I
SampleID
cd31+, tdTomato+ :: Freq. of Parent
FMO tdTomato 0
Tumor VE-TOM mouse 1 86.9
Tumor VE-TOM mouse 2 97.4
Tumor VE-TOM mouse 3 97.8

Figure EV1.

Figure EV2. Time-lapse intravital microscopy of B16F10 tumor in VE-TOM mice.

A–C The developing tumor vasculature was imaged over time in an abdominal window chamber model with two-photon microscopy with (A) GFP-labeled tumor cells, (B) TdTomato-labeled TECs in cyan, and (C) perfusion.

D–M The following parameters were quantified from the segmented image each day: (D) vessel tortuosity, (E) directional coherence, (F) perfusion, (G) number of sprouts, (H) fraction of branches <80 μm, (I) fraction of branches >400 μm, (J) length-to-diameter ratio, (K) nodes per mm^2, (L) vessel length, and (M) vessel diameter.

N Average diameter of perfused to non-perfused vessel in MC38 tumors.

Data information: Data represent mean ± SD, $n = 3$ biological replicates; $^*P < 0.05$, $^{**}P < 0.01$ by one-way analysis of variance with multiple comparisons (ANOVA). Scale bar in (A), (B), and (C): 1 mm.

 © 2022 The Authors

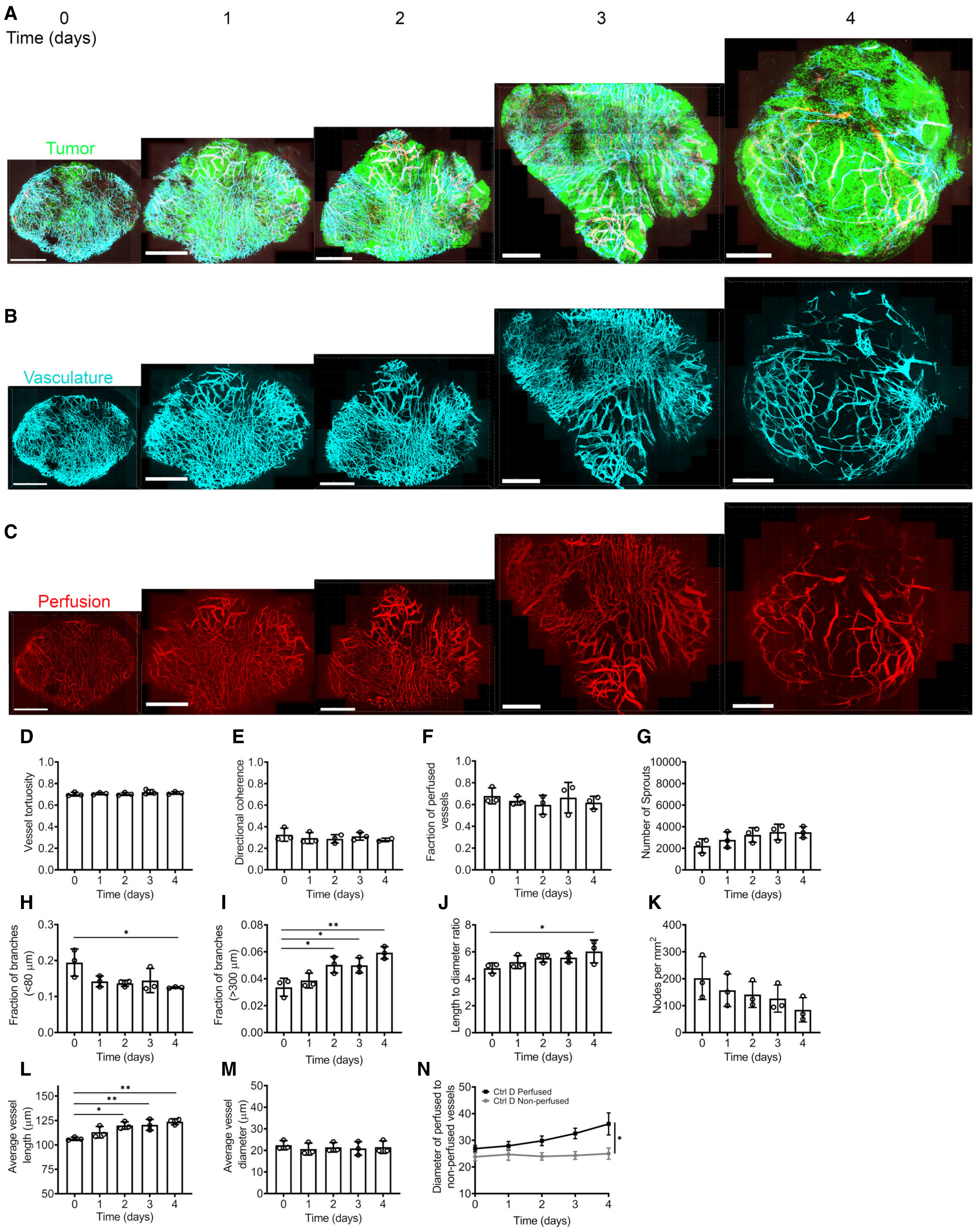
A
Time (days)
0
1
2
3
4
Tumor
B
Vasculature
C
Perfusion
D
Vessel tortuosity
Time (days)
E
Directional coherence
F
Faction of perfused vessels
G
Number of Sprouts
H
Fraction of branches (<80 μm)
I
Fraction of branches (>300 μm)
J
Length to diameter ratio
K
Nodes per mm2
L
Average vessel length (μm)
M
Average vessel diameter (μm)
N
Diameter of perfused to non-perfused vessels
Ctrl D Perfused
Ctrl D Non-perfused

Figure EV2.

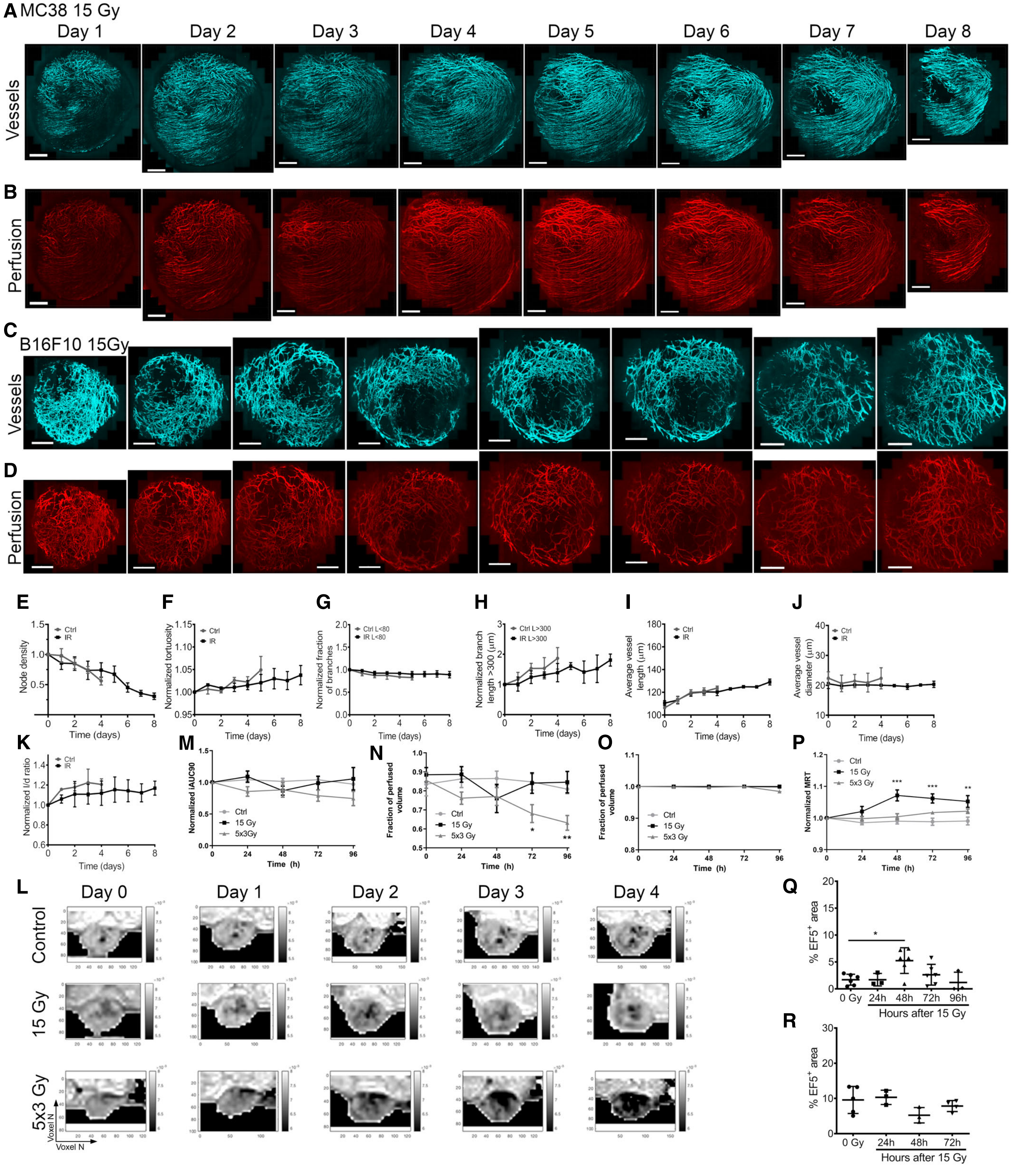

Figure EV3.

© 2022 The Authors

◀ **Figure EV3. Time-lapse intravital microscopy of MC38 and B16F10 tumors of VE-TOM mice, DCE-MRI imaging of MC38 tumors and EF5 analysis in MC38 and B16F10 tumors.**

A, B Timeline of MC38 tumor vasculature (cyan, A) and perfusion (red, B) after single dose of 15 Gy.

C, D Timeline of B16F10 tumor vasculature (cyan, C) and perfusion (red, D) after single dose of 15 Gy IR.

E–K Quantified B16F10 tumor vascular parameters, for each day of imaging the following parameters were quantified from segmented images and normalized to day one if indicated: (E) normalized node density, (F) normalized tortuosity, (G) normalized branch length < 80 μm, (H) normalized branch length > 300 μm, (I) vessel branch length, (J) vessel diameter, and (K) normalized length-to-diameter ratio.

L–P DCE-MRI imaging and quantification of subcutaneous MC38 tumors. Representative DCE-MRI images of subcutaneous MC38 tumors injected with gadolinium-based contrast agent (L). Initial area under the curve (first 90 s after contrast agent injection) iAUC90 was quantified and normalized to day 0 (M). Fraction of perfused tumor volume at 90 s after contrast agent injection (N). Fraction of perfused tumor volume at the end of the imaging session (O). Mean residence time of the contrast agent (P).

Q, R Quantification of percent of $EF5^+$ tumor area in MC38 tumors (Q) and B16F10 tumors (R) after irradiation.

Data information: Data represent mean ± SEM, *n* = 3–4 biological replicates in (E–K), *n* = 6–7 biological replicates in (M–P), *n* = 3–6 biological replicates in (Q, R). $^*P < 0.05$, $^{**}P < 0.01$ by analysis of variance (ANOVA). Scale bars: in (A–D) 1 mm. Color scale in (L) represents the signal enhancement in DCE-MRI images due to the presence of gadolinium-based contrast agent.

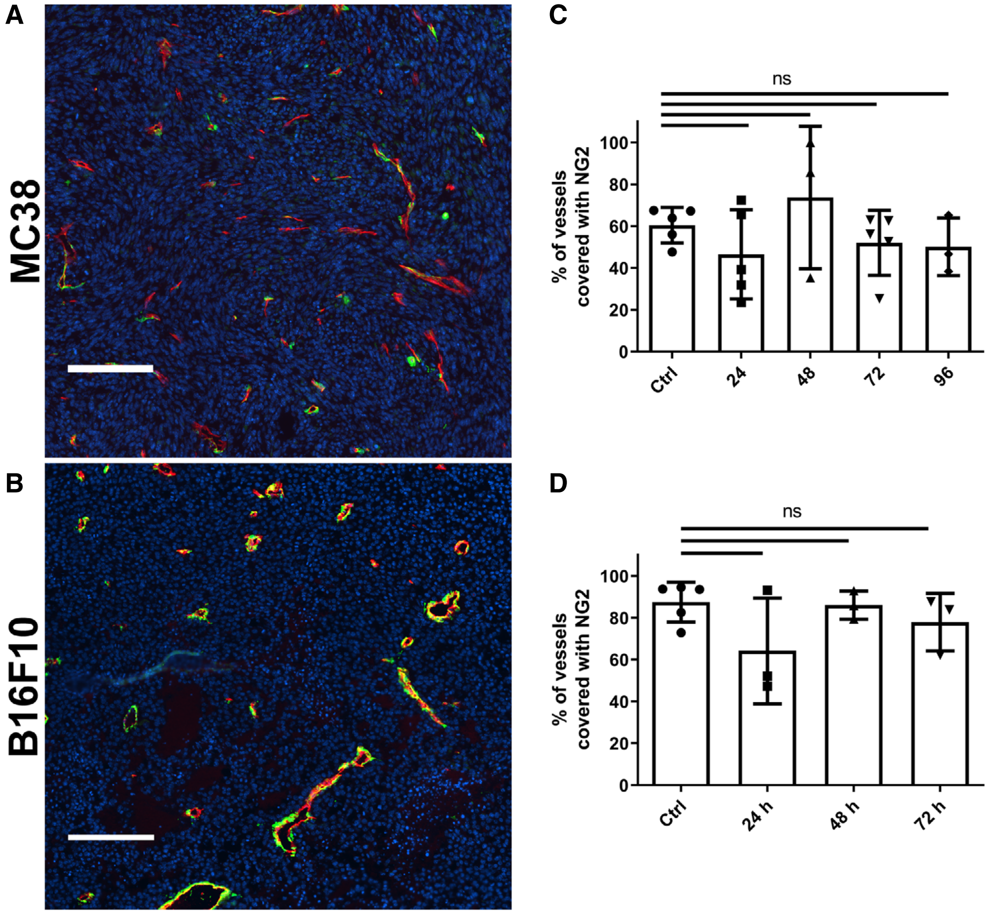

Figure EV4. Co-staining of tumor blood vessels with NG2 and CD31.

A, B Representative immunofluorescent micrographs of control and treated tumor vasculature from (A) MC38 tumors and (B) B16F10 tumors stained with anti-CD31 (red), anti-NG2 (green) and counterstained with Hoechst (blue).

C, D Quantification of vessel coverage with NG2 after single dose of 15 Gy IR from immunofluorescent images of whole (C) MC38 tumor (*n* = 3–5 biological replicates per group) or (D) B16F10 tumor sections (*n* = 3–5 biological replicates per group).

Data information: Scale bar: 100 μm. Data represent mean ± SD. ns—$P > 0.05$ by one-way analysis of variance with multiple comparisons (ANOVA).

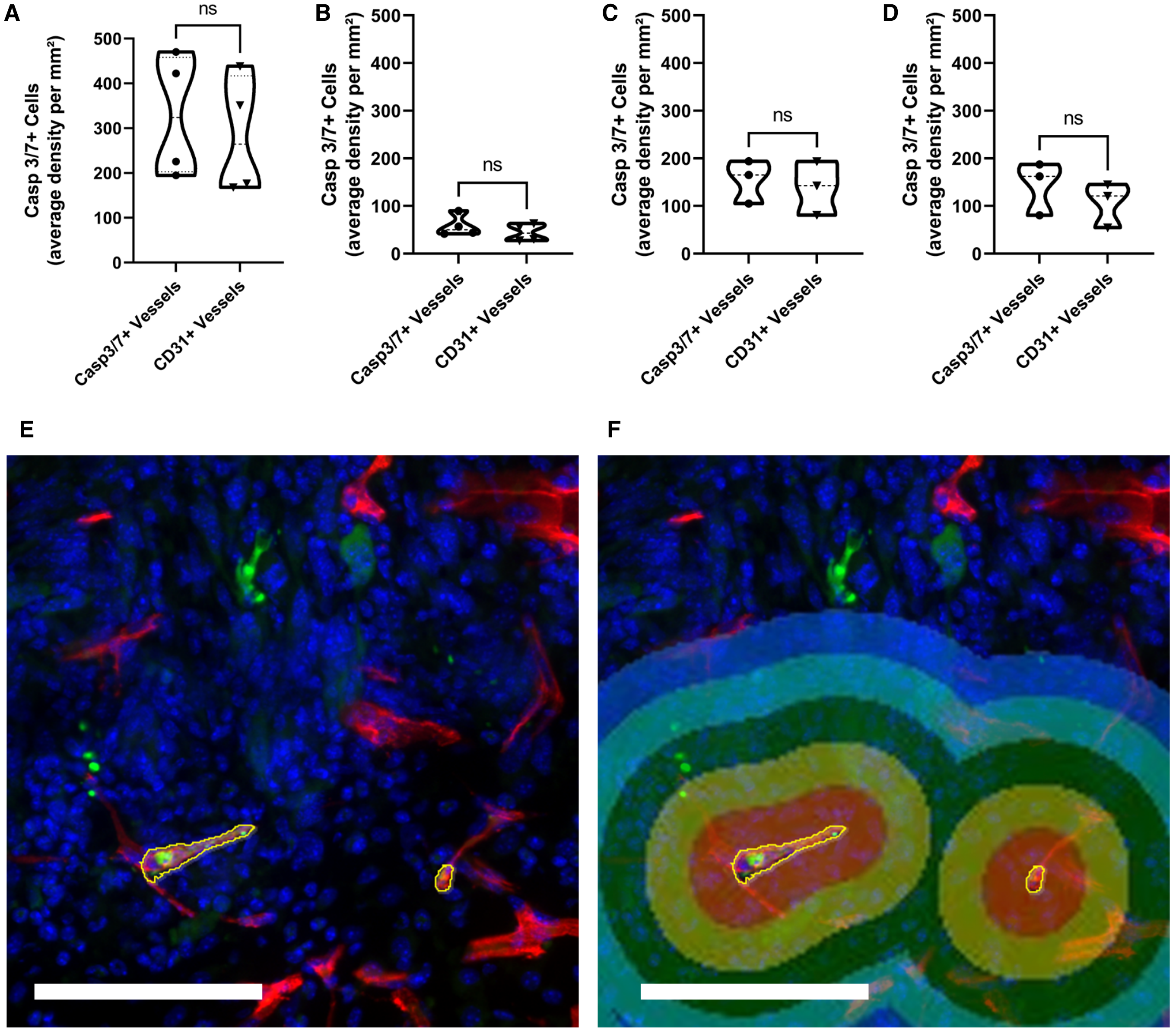

Figure EV5. Average density of Caspase-3/7-positive cells close to blood vessels.

A–F The density per mm^2 of Caspase-3/7 (Casp 3/7)-positive cells within 100 μm radius from (Casp 3/7)-positive TECs was determined at 48 h after single-dose irradiation to (A) MC38 tumors, (B) B16F10 tumors or (C) 48 h and (D) 168 h after the first dose of 5x 3 Gy of fractionated irradiation in MC38 tumors. A representative image of (E) MC38 tumor vasculature with Casp 3/7-positive TEC and other cells with the (F) overlaid analysis mask. Tumor sections were stained with anti-CD31 (red), anti-Cas 3/7 (green) and counterstained with Hoechst (blue). Scale bar: 100 μm.

Data information: Presented are violin plots of all data with dashed line representing median and dotted lines quartiles. n = 3–4 biological replicates per group. ns—P > 0.05 by one-way analysis of variance with multiple comparisons (ANOVA).

 © 2022 The Authors
