# Supplementary material for: Endothelial cell death after ionizing radiation does not impair vascular structure in mouse tumor models
Source: EMBO Rep. 2022 Jul 18;23(9):e53221. doi: 10.15252/embr.202153221 (PMC9442312; doi:10.15252/embr.202153221)
Supplement: Supplementary file 3 [file EMBR-23-e53221-s003.pdf]

EMBO reports

Endothelial cell death after ionizing radiation does not impair vascular structure in mouse tumor models

Jakob Kaeppler, Jianzhou Chen, Mario Buono, Jenny Vermeer, Pavitra Kannan, Wei-Chen Cheng, Dimitris Voukantsis, James Thompson, Mark Hill, Danny Allen, Ana Gomes, Veerle Kersemans, Paul Kinchesh, Sean Smart, Francesca Buffa, Claus Nerlov, Ruth Muschel, and Bostjan Markelc
DOI: 10.15252/embr.202153221

Corresponding author(s): Bostjan Markelc (bmarkelc@onko-i.si) , Ruth Muschel (ruth.muschel@oncology.ox.ac.uk)

Review Timeline:

Submission Date:	7th May 21
Editorial Decision:	17th Jun 21
Revision Received:	15th Dec 21
Editorial Decision:	8th Mar 22
Revision Received:	15th May 22
Accepted:	27th Jun 22

Editor: Martina Rembold

Transaction Report:

(Note: With the exception of the correction of typographical or spelling errors that could be a source of ambiguity, letters and reports are not edited. Depending on transfer agreements, referee reports obtained elsewhere may or may not be included in this compilation. Referee reports are anonymous unless the Referee chooses to sign their reports.)

1st Editorial Decision 17th Jun 2021

Dear Dr. Markelc

Thank you for the submission of your research manuscript to our journal. We have now received the full set of referee reports that is copied below.

As you will see, the referees acknowledge that the findings are potentially interesting. However, they also point out some technical concerns and have a number of suggestions for how the study should be strengthened, and I think that all of them should be addressed.

Given these constructive comments, we would like to invite you to revise your manuscript with the understanding that the referee concerns (as detailed above and in their reports) must be fully addressed and their suggestions taken on board. Please address all referee concerns in a complete point-by-point response. Acceptance of the manuscript will depend on a positive outcome of a second round of review. It is EMBO reports policy to allow a single round of revision only and acceptance or rejection of the manuscript will therefore depend on the completeness of your responses included in the next, final version of the manuscript.

We invite you to submit your manuscript within three months of a request for revision. This would be September 17th in your case. However, we are aware of the fact that many laboratories are not fully functional due to COVID-19 related shutdowns and we have therefore extended the revision time for all research manuscripts under our scooping protection to allow for the extra time required to address essential experimental issues. Please contact us to discuss the time needed and the revisions further.

***IMPORTANT NOTE: we perform an initial quality control of all revised manuscripts before re-review. Your manuscript will FAIL this control and the handling will be DELAYED if the following APPLIES:

1) A data availability section is missing.
2) Your manuscript contains error bars based on n=2. Please use scatter blots showing the individual datapoints in these cases. The use of statistical tests needs to be justified.

When submitting your revised manuscript, please carefully review the instructions that follow below. Failure to include requested items will delay the evaluation of your revision.***

When submitting your revised manuscript, we will require:

1) a .docx formatted version of the manuscript text (including legends for main figures, EV figures and tables). Please make sure that the changes are highlighted to be clearly visible.
Please also note that all materials and methods must be part of the main manuscript and should not be in the Supplement.

2) individual production quality figure files as .eps, .tif, .jpg (one file per figure).
Please download our Figure Preparation Guidelines (figure preparation pdf) from our Author Guidelines pages https://www.embopress.org/page/journal/14693178/authorguide for more info on how to prepare your figures.

3) a .docx formatted letter INCLUDING the reviewers' reports and your detailed point-by-point responses to their comments. As part of the EMBO Press transparent editorial process, the point-by-point response is part of the Review Process File (RPF), which will be published alongside your paper.

4) a complete author checklist, which you can download from our author guidelines (). Please insert information in the checklist that is also reflected in the manuscript. The completed author checklist will also be part of the RPF.

5) Please note that all corresponding authors are required to supply an ORCID ID for their name upon submission of a revised manuscript (). Please find instructions on how to link your ORCID ID to your account in our manuscript tracking system in our Author guidelines
()

6) We replaced Supplementary Information with Expanded View (EV) Figures and Tables that are collapsible/expandable online. A maximum of 5 EV Figures can be typeset. EV Figures should be cited as 'Figure EV1, Figure EV2" etc... in the text and their respective legends should be included in the main text after the legends of regular figures.

- For the figures that you do NOT wish to display as Expanded View figures, they should be bundled together with their legends in a single PDF file called *Appendix*, which should start with a short Table of Content. Appendix figures should be referred to in the main text as: "Appendix Figure S1, Appendix Figure S2" etc. See detailed instructions regarding expanded view here:

- Additional Tables/Datasets should be labeled and referred to as Table EV1, Dataset EV1, etc. Legends have to be provided in a separate tab in case of .xls files. Alternatively, the legend can be supplied as a separate text file (README) and zipped together with the Table/Dataset file.

7) Please list the accession numbers and database for the RNAseq data in a formal "Data Availability " section (placed after Materials & Method) that follows the model below (see also < https://www.embopress.org/page/journal/14693178/authorguide#dataavailability>). Please note that the Data Availability Section is restricted to new primary data that are part of this study.

Data availability

The datasets (and computer code) produced in this study are available in the following databases:

- RNA-Seq data: Gene Expression Omnibus GSE46843 (https://www.ncbi.nlm.nih.gov/geo/query/acc.cgi?acc=GSE46843)
- [data type]: [name of the resource] [accession number/identifier/doi] ([URL or identifiers.org/DATABASE:ACCESSION])

*** Note - All links should resolve to a page where the data can be accessed. ***

8) We would also encourage you to include the source data for figure panels that show essential data. Numerical data should be provided as individual .xls or .csv files (including a tab describing the data). For blots or microscopy, uncropped images should be submitted (using a zip archive if multiple images need to be supplied for one panel). Additional information on source data and instruction on how to label the files are available .

9) Regarding data quantification
The following points MUST be specified in each figure legend:
- the name of the statistical test used to generate error bars and P values,
- the number (n) of independent experiments (please specify technical or biological replicates) underlying each data point,
- the nature of the bars and error bars (s.d., s.e.m.)

Discussion of statistical methodology can be reported in the materials and methods section, but figure legends should contain a basic description of n, P and the test applied.
- Please also include scale bars in all microscopy images.

10) Our journal encourages inclusion of *data citations in the reference list* to directly cite datasets that were re-used and obtained from public databases. Data citations in the article text are distinct from normal bibliographical citations and should directly link to the database records from which the data can be accessed. In the main text, data citations are formatted as follows: "Data ref: Smith et al, 2001" or "Data ref: NCBI Sequence Read Archive PRJNA342805, 2017". In the Reference list, data citations must be labeled with "[DATASET]". A data reference must provide the database name, accession number/identifiers and a resolvable link to the landing page from which the data can be accessed at the end of the reference. Further instructions are available at .

11) As part of the EMBO publication's Transparent Editorial Process, EMBO reports publishes online a Review Process File to accompany accepted manuscripts. This File will be published in conjunction with your paper and will include the referee reports, your point-by-point response and all pertinent correspondence relating to the manuscript.

You are able to opt out of this by letting the editorial office know (emboreports@embo.org). If you do opt out, the Review Process File link will point to the following statement: "No Review Process File is available with this article, as the authors have chosen not to make the review process public in this case."

We would also welcome the submission of cover suggestions, or motifs to be used by our Graphics Illustrator in designing a cover.

I look forward to seeing a revised form of your manuscript when it is ready. Please use this link to submit your revision:
https://embor.msubmit.net/cgi-bin/main.plex

Yours sincerely

Martina Rembold, PhD
Senior Editor
EMBO reports

****

Referee #1:

Kaeppler et al. examined the effects of ionizing radiation on tumor vascular structure using two murine non-orthotopic transplant tumor models in mice with tdTomato labeled endothelial cells. The authors used the highly vascularized MC38 colon cancer model and the moderately vascularized B16F10 melanoma model. GFP-labeled tumor cells and endothelial cells were visualized using multiphoton microscopy in an abdominal window chamber model. Tumor bearing mice were irradiated with 15 Gy or 5 x 3 Gy and the vascular structures in the tumors were evaluated. RNAseq data were also collected from the tumor endothelial cells. The authors showed that in these 2 models radiation does not impair overall tumor vascular structure. The topic of this manuscript is of broad interest, the text is well written, and the data provide an advance to the field. A few modifications will further strengthen the manuscript:

Major Comments

1. The data demonstrate that the endothelial cells of smaller or non-perfused vessels were positive for Caspase 3 and more susceptible to radiation-induced apoptosis than larger perfused vessels. The authors do not propose a mechanism for the difference in sensitivity of the small vs. large vessels. The authors should graph the EdU data (Figure 4) from the unirradiated tumors stratified by small and large vessels to determine if the smaller vessels are more proliferative at baseline, which could therefore provide a potential rationale for why they are more sensitive to radiation induced apoptosis.

2. Do the authors have data to address whether there is indirect tumor cell death as a consequence of vascular cell death? Are the dying tumor cells next to the small vessels that are dying or is the tumor cell death independent of the vascular cell death? Several publications have claimed that indirect cell death of tumor cells as a consequence of radiation-induced vascular damage is an important mechanism for tumor response to radiation (Song et al., IJROBP 2016). Although not essential for the main conclusions of this manuscript, addressing this point with data or at least in the discussion would be of interest to the field.

3. The Moding et al. (Int J Radiat Oncol Biol Phys, 2013) manuscript, that the authors cited, demonstrated increased vascular permeability after irradiation using fluorescently labeled dextran accumulation. The authors should compare and contrast their data using the Qdots to track perfusion with these published data from Moding et al. Is there evidence of vascular leakiness in the 2 models that have been studied?

Minor comments

1. Panels S4 C and D should be moved to the main figures.
2. Panels S3 A and B should be moved to the main figures.
3. Page 5 after 'Park et al, 2012' there is an extra parenthesis.

Referee #2:

Kaeppler and colleagues performed multiphoton microscopy in two murine tumor models exposed to either a single (15 Gy) or fractionated (5x3 Gy) dose(s). They report that perfused vasculature is barelay altered while proliferation of angiogenic endothelial cells is impaired.
This is a sound study that bring a new angle to the long unresolved issue of the effecst of radiotherapy on the tumor vasculature.

This study is technically sound and the authors's claims are generally supported by the data.
Still a concern is the nature of the perfused blood vessels. Even though perfusion may indicate mature blood vessels, mouse models with highly aggressive generation of a neovasculature (even in B16 melanoma) are far to reflect the human situation where tumors develop much more slowly. Alpha-SMA staining for instance could be used to further segregate mature and immature, perfused blood vessels.
A second concern is related to gene expression analysis (incl scRNAseq) that are performed on FACsorted TEC. The relevance of mRNA changes is questionable considering processing time and the focus on single 48h post IR time. Information on the alpha-SMA coverage of the irradiated vasculature and the extent of VEGF production in response to IR would provide more insightful sets of data for the community working in this field. The impact of inhibitors of VEGF signaling on the read-outs used by the authors could also be useful to determine the extent of (basal and induced) protection offered by the growth factor.

Referee #3:

Criticisms Kaeppler

Kaeppler et al. studies the effect of irradiation on the vasculature in two tumor models, MC38 and B16F10, both grown as subcutaneous tumors in C57Bl/6 mice and irradiated or not. Tumor vessel properties are followed through an abdominal window. The authors ask how radiation therapy on the one hand allows increased oxygenation and perfusion and on the other hand, leads to endothelial cell apoptosis. They show that different vascular pools are differently affected by irradiation. While small, nonperfused vessels are specifically targeted to undergo apoptosis, larger vessels are unaffected by the irradiation and increase their diameter further during the course of the treatment.

Even though prior literature has shown that irradiation may lead to tumor vascular remodelling and transient normalization, this careful characterization of different vascular populations has not been done before.

1. The authors use Qdots to score the degree of vessel perfusion and find that small vessels are not perfused. The challenge is obviously to efficiently perfuse the vasculature and in a sensitive manner detect also perfusion of small vessels. As a control, this reviewer suggests to show that 100% of small vessels {less than or equal to} 10 micrometer are perfused in a healthy organ.
2. The authors show that perfusion increases very considerably in particular with a single dose compared to fractionated irradiation (FIR) (Fig. 3D). In Fig. S3P however, perfusion decreases clearly in FIR-treated tumors while single dose IR remains unaffected. Please explain. Use the same designations in the figures throughout.
3. Bulk RNAseq on isolated TECs is presented in Fig. 5. Several of the panels such as D-F can be moved to the supplement as there is no effect of irradiation on gene expression. The two panels showing affected pathway are redundant; one is enough. In the single cell RNAseq it is surprising that arterial gene expression decreases with irradiation, as arterials are bigger vessels which one would expect to be perfused and that smaller vessels {less than or equal to} 10 micrometer would be capillaries? Please explain.

Referee #1:

Kaeppler et al. examined the effects of ionizing radiation on tumor vascular structure using two murine non-orthotopic transplant tumor models in mice with tdTomato labeled endothelial cells. The authors used the highly vascularized MC38 colon cancer model and the moderately vascularized B16F10 melanoma model. GFP-labeled tumor cells and endothelial cells were visualized using multiphoton microscopy in an abdominal window chamber model. Tumor bearing mice were irradiated with 15 Gy or 5 x 3 Gy and the vascular structures in the tumors were evaluated. RNAseq data were also collected from the tumor endothelial cells. The authors showed that in these 2 models radiation does not impair overall tumor vascular structure. The topic of this manuscript is of broad interest, the text is well written, and the data provide an advance to the field. A few modifications will further strengthen the manuscript:

Major Comments

1. The data demonstrate that the endothelial cells of smaller or non-perfused vessels were positive for Caspase 3 and more susceptible to radiation-induced apoptosis than larger perfused vessels. The authors do not propose a mechanism for the difference in sensitivity of the small vs. large vessels. The authors should graph the EdU data (Figure 4) from the unirradiated tumors stratified by small and large vessels to determine if the smaller vessels are more proliferative at baseline, which could therefore provide a potential rationale for why they are more sensitive to radiation induced apoptosis.

We thank the reviewer for the suggestion. We have now graphed the EdU data stratified by small and large vessels and indeed the small vessels were more proliferative in both tumor models, which could be a potential rationale for their higher radiosensitivity. We have added panels R and S to Figure 4 showing this data. Additionally we have modified the text to reflect the new data (Page 14):

"*Interestingly, when we compared the number of EdU positive TECs in control non-irradiated tumors, we observed that the smaller vessels are more proliferative than large vessels in both tumor models (Figure 4T, U) which could also explain the more prominent apoptosis observed in small vessels after IR (Figure H, I).*"

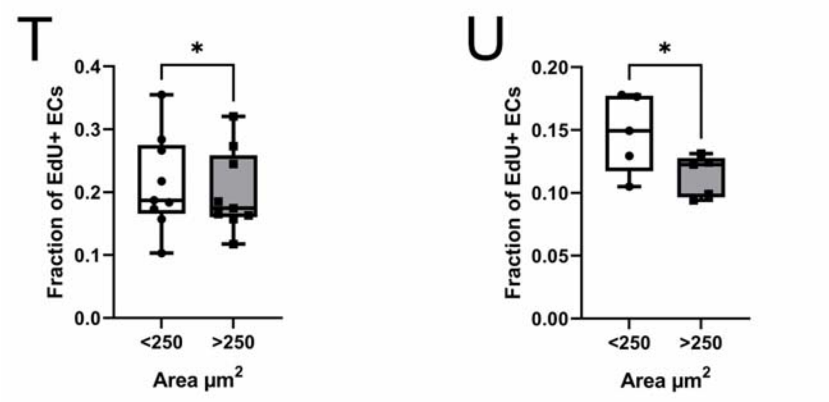

… Quantification of proliferating TECs in **T)** MC38 tumors (n= 9) and **(U)** B16F10 tumors (n=5) in small (area <250 μm^2) and large (area <250 μm^2) tumor blood vessels. *< 0.05 by paired t-test. …

2. Do the authors have data to address whether there is indirect tumor cell death as a consequence of vascular cell death? Are the dying tumor cells next to the small vessels that are dying or is the tumor cell death independent of the vascular cell death? Several publications have claimed that indirect cell death of tumor cells as a consequence of radiation-induced vascular damage is an important mechanism for tumor response to radiation (Song et al., IJROBP 2016). Although not essential for the main conclusions of this manuscript, addressing this point with data or at least in the discussion would be of interest to the field.

We thank the reviewer for pointing out this phenomenon. We have now analysed the average density of Caspase 3/7+ cells in 100 µm radius of Caspase 3/7+ vessels at 48h after irradiation. We chose this time point because it is the time when we have observed the highest percent of Caspase 3/7+ tumor cells (Fig S4, Biii) after a single dose of XRT. For fractionated XRT we have additionally performed the analysis at 168h after the first dose of XRT. Using this analysis, we were not able to confirm indirect tumor cell death due to vascular cell death. We have added a new figure (Fig S6) with the new data and modified the text to reflect the new data (Page 13):

"Moreover, the death of TECs did not result in increased apoptosis of adjacent tumor cells, as the average density of caspase-3 positive cells was not significantly increased within 100 µm of the caspase-3 positive TEC compared to those adjacent to the viable vasculature (Figure S6-F)."

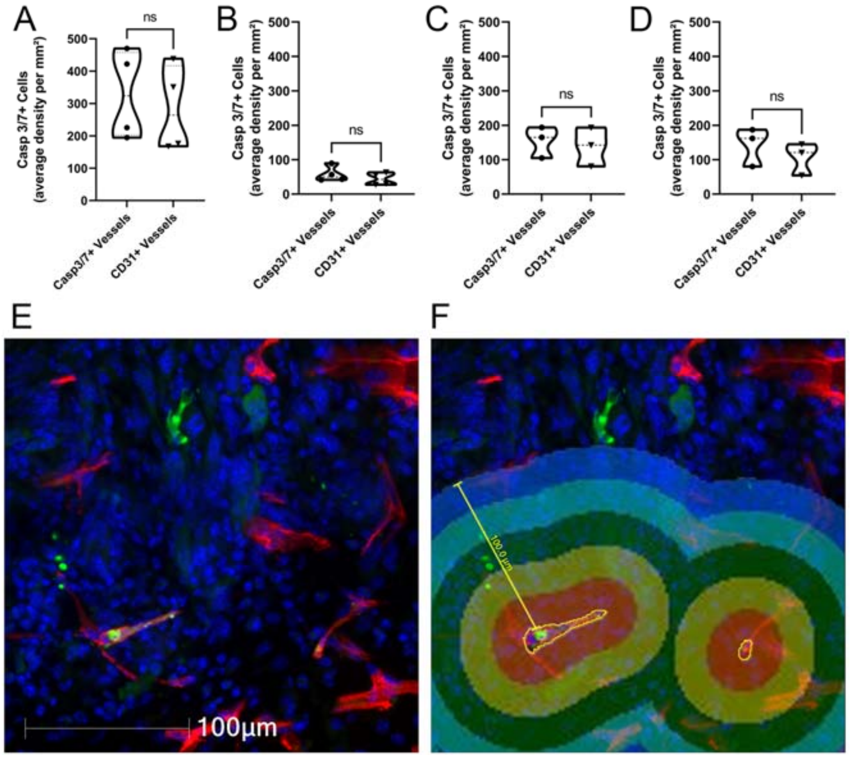

Fig. S6. Average density of Caspase 3/7 positive cells close to blood vessels. The density per mm^2 of Caspase 3/7 (Casp 3/7) positive cells within 100 µm radius from (Casp 3/7) positive TECs was determined at 48h after single dose irradiation to **(A)** MC38 tumors, **(B)** B16F10 tumors or **(C)** 48h and **(D)** 168h after the first dose of 5x 3 Gy of fractionated irradiation in MC38 tumors. A representative image of **(E)** MC38 tumor vasculature with

Casp 3/7 positive TEC and other cells with the **(F)** overlaid analysis mask. Tumor sections were stained with anti-CD31 (red), anti - Cas 3/7 (green) and counterstained with Hoechst (blue). Scale bar: 100 µm. Presented are violin plots of all data. n= 3-4 per group. ns – $P > 0.05$ by one-way analysis of variance with multiple comparisons (ANOVA).

3. The Moding et al. (Int J Radiat Oncol Biol Phys, 2013) manuscript, that the authors cited, demonstrated increased vascular permeability after irradiation using fluorescently labeled dextran accumulation. The authors should compare and contrast their data using the Qdots to track perfusion with these published data from Moding et al. Is there evidence of vascular leakiness in the 2 models that have been studied?

The study by Moding et al (Moding *et al*, 2013) showed increased accumulation of 2 MDa fluorescently labelled dextrans on frozen tissue section and a liposomal-iodinated contrast agent imaged by dual energy (DE) micro-computed tomography (micro-CT) in a mouse model of soft tissue sarcoma after a single dose irradiation of 20 Gy. Whereas in our study we used two different tumor models, MC38 colon adenocarcinoma and B16F10 melanoma and a lower single dose of irradiation (i.e. 15 Gy) or fractionated irradiation with similar BED (i.e. 5x 3 Gy). The permeability of tumor vasculature in different tumor models varies significantly, and also dependent on the size of particle used to measure the permeability as well as on other properties such as shape and charge (Smith *et al*, 2012; Dreher *et al*, 2006). Moreover, the use of a higher dose of irradiation in the Moding et al study could also be responsible for the observed vascular permeability. In our study, using Qdots 705 (REF), which are larger in comparison to 2 MDa dextrans (Armstrong *et al*, 2004), but smaller than the liposomal-iodinated contrast agent used in the Moding et al study, we did not observe their extravasation or accumulation in the tumors after irradiation (Fig S3). We agree that for a direct comparison of changes in vascular permeability after irradiation the use of fluorescently labelled dextrans of similar or different sizes would be needed, however, as the focus of the study was to image the same tumor for several days, we have intentionally avoided fluorescently labelled dextrans, or other particles, that readily extravasate and accumulate in the tumor as they then impede imaging on later time-points. Moreover, Qdot's (Qtracker® non-targeted quantum dots, Thermo Fischer Scientific) do not extravasate readily and have a long plasma half-life in comparison to fluorescently labelled dextrans. We have also stated this in our manuscript in the result section (Page 11):

"*No indication of vascular rupture based on leakage of Qdots was observed (Figure 3A-C) and no other evidence of compromise of functional vascular structure was evident (Figure S3C, D).".* We have now added an additional paragraph describing this in the Discussion.

Interestingly, we did not observe an increase in the permeability of tumor blood vessels for Qdot's 705 after irradiation in our imaging experiments (Fig 3 and S3). There are contrary reports of increased permeability of tumor blood vessels after irradiation (Moding et al, 2013). As our tumor models and contrast agent used, as well as the dose of irradiation that was delivered, differ from the ones in Moding et al, the results are not directly comparable. The measured change in the permeability of tumor vasculature depends on the tumor model, the size and type of particles used to determine the permeability and also on their shape. Thus, direct comparisons between studies are only possible when at least the same particles are used for determining the permeability of tumor vasculature. This is also one of the drawbacks of this study, as we have intentionally opted for Qdot's (Qtracker® non-targeted quantum dots, Thermo Fischer Scientific), that do not extravasate readily and have a long

plasma half-life in comparison to fluorescently labelled dextrans. This choice enabled us to perform daily imaging of the same tumor, however; as a result we lost the ability to directly compare the changes in the permeability of tumor blood vessels after irradiation to other published studies..”

Minor comments

1. Panels S4 C and D should be moved to the main figures.

- We moved panels C and D from S4 to the main figure 4 (now panels F, G)

2. Panels S3 A and B should be moved to the main figures.

- We moved panels A and B from S3 to the main figure 3 (now panels M, N)

3. Page 5 after 'Park et al, 2012' there is an extra parenthesis.

- Corrected

Referee #2:

Kaeppler and colleagues performed multiphoton microscopy in two murine tumor models exposed to either a single (15 Gy) or fractionated (5x3 Gy) dose(s). They report that perfused vasculature is barelay altered while proliferation of angiogenic endothelial cells is impaired.
This is a sound study that bring a new angle to the long unresolved issue of the effects of radiotherapy on the tumor vasculature.

This study is technically sound and the authors's claims are generally supported by the data. Still a concern is the nature of the perfused blood vessels. Even though perfusion may indicate mature blood vessels, mouse models with highly aggressive generation of a neovasculature (even in B16 melanoma) are far to reflect the human situation where tumors develop much more slowly. Alpha-SMA staining for instance could be used to further segregate mature and immature, perfused blood vessels.

We agree with the reviewer that mouse models with highly aggressive generation of neovasculature, like MC38 and B16F10 that we used in our study, do not really reflect the situation in many human tumors. Thus, we have avoided drawing direct comparison to clinical situations in our paper. In our manuscript, we have also limited our designation of observed blood vessels to perfused and non-perfused ones, as we were unable to incorporate additional fluorescence signal into our imaging setup to further differentiate between different types of blood vessels in the tumor (one option would be to stain for a-SMA as per reviewers suggestion). However, we have now stained frozen sections for CD31 and NG2 as a common marker for pericytes. We have chosen NG2 instead of alpha-SMA, which are both commonly used pericyte markers, due to the long-term experience and already established staining protocol for this antibody in our lab. Although even with this data we cannot differentiate between the mature perfused and immature perfused blood vessels, we have now shown that the single dose of irradiation of 15 Gy does not modify the pericyte coverage of tumor blood vessels in either the MC38 or B16F10 tumor model. We have prepared a new figure with this data (Fig S4) and added the pertinent information to the manuscript (Page 12):

“Lastly, we have co-stained tumor section for NG2 proteoglycan and CD31 to determine whether irradiation effects the pericyte coverage of tumor blood vessels; however, we did not observe any nor in MC38 or B16F10 tumor model (Figure S4).”

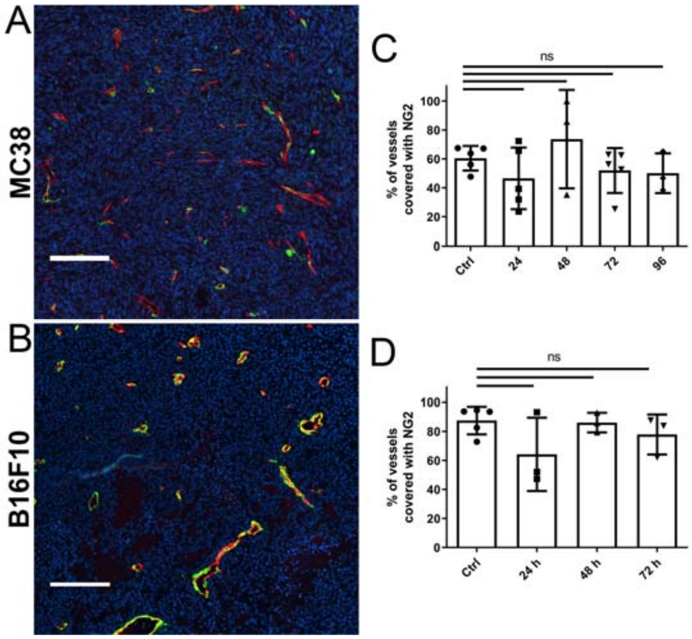

Fig. S4. Co-staining of tumor blood vessels with NG2 and CD31. Representative **i**mmunofluorescent micrographs of control and treated tumor vasculature from **(A)** MC38 tumors and **(B)** B16F10 tumors stained with anti-CD31 (red), anti-NG2 (green) and counterstained with Hoechst (blue). Quantification of apoptotic vessels after single dose 15 Gy IR from immunofluorescent images of whole **(C)** MC38 tumor (n= 3-5 per group) or **(D)** B16F10 tumor sections (n= 3’5 per group). Scale bar: 100 µm. Error bars represent mean ± SD. ns – P > 0.05 by one-way analysis of variance with multiple comparisons (ANOVA).

A second concern is related to gene expression analysis (incl scRNAseq) that are performed on FACsorted TEC. The relevance of mRNA changes is questionable considering processing time and the focus on single 48h post IR time. Information on the alpha-SMA coverage of the irradiated vasculature and the extent of VEGF production in response to IR would provide more insightful sets of data for the community working in this field. The impact of inhibitors of VEGF signaling on the read-outs used by the authors could also be useful to determine the extent of (basal and induced) protection offered by the growth factor.

We appreciate the concern raised about the relevance of mRNA changes in relation to processing time and the focus on a single 48h post IR time point. We have carefully considered the time point chosen for the RNAseq, and we based it on our data where we showed that apoptosis of TECs and their still ongoing cell-cycle arrest seems to be most prominent at this time point after a single dose of irradiation. Thus, we wanted to investigate the expression of genes before TECs return to their normal state. Indeed, a more comprehensive analysis would result in a much better understanding of the underlying

molecular changes in TECs after irradiation, and we are planning to perform it in future research. However, we believe that even with the scRNAseq analysis performed at a single time point at 48h after irradiation our results reveal important changes in the expression patterns of TECs indicating on their involvement in the recruitment of immune cells and the changing landscape of different TEC subtypes, which was not yet studied before.

Regarding the processing time of the samples, we have adhered to the best of practice protocols for scRNAseq from tumor samples published at the time of the experiments. Moreover, the processing time of samples in our protocols was even shorter than the one in recently published landmark paper “Single-Cell Transcriptome Atlas of Murine Endothelial Cells” (Kalucka *et al*, 2020) and the subsequent protocol papers that describe in detail the procedures for isolating endothelial cells from different tissues (Sokol *et al*, 2021; Dumas *et al*, 2021).

As mentioned above, we have now co-stained tumor section for NG2 proteoglycan and CD31 to whether irradiation effects the pericyte coverage of tumor blood vessels; however, we did not observe any nor in MC38 or B16F10 tumor model (Figure S4).

We also agree with the reviewer that the extent of VEGF production in response to IR is important and would add another level of importance to our results, especially in respect to the proper use and timing of either VEGF or VEGFR inhibitors in combination with irradiation in clinical settings. However, our main objective was to clearly show what are the effects of irradiation on tumor vascular structure and function and to study the underlying mechanisms in the TECs that are responsible for the observed changes.

Referee #3: Criticisms Kaeppler

Kaeppler et al. studies the effect of irradiation on the vasculature in two tumor models, MC38 and B16F10, both grown as subcutaneous tumors in C57Bl/6 mice and irradiated or not. Tumor vessel properties are followed through an abdominal window. The authors ask how radiation therapy on the one hand allows increased oxygenation and perfusion and on the other hand, leads to endothelial cell apoptosis. They show that different vascular pools are differently affected by irradiation. While small, nonperfused vessels are specifically targeted to undergo apoptosis, larger vessels are unaffected by the irradiation and increase their diameter further during the course of the treatment.

Even though prior literature has shown that irradiation may lead to tumor vascular remodelling and transient normalization, this careful characterization of different vascular populations has not been done before.

1. The authors use Qdots to score the degree of vessel perfusion and find that small vessels are not perfused. The challenge is obviously to efficiently perfuse the vasculature and in a sensitive manner detect also perfusion of small vessels. As a control, this reviewer suggests to show that 100% of small vessels {less than or equal to} 10 micrometer are perfused in a healthy organ.

We recognize the importance of independently showing that the imaging agent used in the studies is efficient in perfusing even small vessels; however, in our case this would require us to use a different imaging window covering an organ in live mice. Unfortunately, our animal licence does not allow us to perform any other experiments with the imaging windows except in the setting that it was used in our manuscript. We hope that the plethora of

different organs, imaging modalities and imaging windows where others used Qdots would be sufficient to show that Qdots can perfuse even the smallest vessels. For instance, Qdots were already used to perfuse and image vasculature in mouse liver (Guidotti *et al*, 2015), bone marrow (Brooks *et al*, 2021), urothelium (Sano *et al*, 2016), brain (Sorensen *et al*, 2018) and in tumors (Stroh *et al*, 2005; Smith *et al*, 2012)

2. The authors show that perfusion increases very considerably in particular with a single dose compared to fractionated irradiation (FIR) (Fig. 3D). In Fig. S3P however, perfusion decreases clearly in FIR-treated tumors while single dose IR remains unaffected. Please explain. Use the same designations in the figures throughout.

In figure 3D we showed the percent of perfused vessels in the intravital images, whereas in Figure S3P (now S3N) we showed the fraction of initially perfused tumor volume determined by DCE-MRI. These two parameters are not necessarily connected as the data in figure S3N shows how quickly the tumor blood vessels perfuse the tumor immediately after the injection of the DCE-MRI contrast agent, whereas the data in figure 3D shows all the blood vessels that were perfused in the tumor during the intravital imaging sessions. A better comparison would be to compare the data in Figure 3D with the data in Figure S3O (previously Q) that shows the total perfused tumor volume at the end of the DCE-MRI imaging sessions. As evident from the graph, even after 5x3 Gy the total perfused tumor volume does not change compared to control tumors. Thus, we believe that there is a higher fraction of perfused blood vessels after irradiation, because some of the non-functional blood vessels are destroyed and this can be captured only on the level of single blood vessels with intravital imaging (Figure 3D). But they may be unable to be as rapidly perfused as before irradiation (which is captured in Figure S3N). However, the entire tumor volume is still perfused in the end (Figure S3O).

3. Bulk RNAseq on isolated TECs is presented in Fig. 5. Several of the panels such as D-F can be moved to the supplement as there is no effect of irradiation on gene expression. The two panels showing affected pathway are redundant; one is enough. In the single cell RNAseq it is surprising that arterial gene expression decreases with irradiation, as arterials are bigger vessels which one would expect to be perfused and that smaller vessels {less than or equal to} 10 micrometer would be capillaries? Please explain.

We have modified the Figure 5 according to the reviewer's comments.

We agree with the reviewer that one would expect that bigger vessels would be arteries and that they would remain after irradiation. However, from our data and from data of others (Zhao *et al*, 2018), it seems that majority of tumor endothelial cells, at least in murine tumor models, show a "capillary" or "transition" phenotype and are neither fully arterial or venule like. Thus, although the vessels act as arteries, venules, capillaries, their gene expression signature in the tumor does not recapitulate that. This is especially striking in case of "venules", this type of EC phenotype was almost absent even in the control tumors. Thus, we believe that in our case these "phenotypes" are not directly connected to vessel diameter, but more to the ECs becoming tumor ECs and not regular ECs. Further evidence on the problems with obtaining/using defined gene signatures for different subtypes of blood vessels even in normal tissues were recently published "*Single-Cell Transcriptome Atlas of Murine Endothelial Cells*" and "*Endothelial cell plasticity at the single-cell level*" (Kalucka *et al*, 2020; Pasut *et al*, 2021). This is even truer for tumor ECs where a robust gene signature for well-known normal EC subtypes was not yet determined. We believe that our results

warrant further research exactly into this problem; what are the EC subtypes in the tumor, what are their expression signatures, and in the case of irradiation how does irradiation modify these subtypes and how could they be exploited to improve radiotherapy.

Armstrong JK, Wenby RB, Meiselman HJ & Fisher TC (2004) The hydrodynamic radii of macromolecules and their effect on red blood cell aggregation. *Biophys J* 87: 4259–4270

Brooks J, Kumar B, Zuro DM, Raybuck JD, Madabushi SS, Vishwasrao P, Parra LE, Kortylewski M, Armstrong B, Froelich J, *et al* (2021) Biophysical Characterization of the Leukemic Bone Marrow Vasculature Reveals Benefits of Neoadjuvant Low-Dose Radiation Therapy. *Int J Radiat Oncol Biol Phys* 109: 60–72

Dreher MR, Liu W, Michelich CR, Dewhirst MW, Yuan F & Chilkoti A (2006) Tumor vascular permeability, accumulation, and penetration of macromolecular drug carriers. *J Natl Cancer Inst* 98: 335–344

Dumas SJ, Meta E, Conchinha N V., Sokol L, Chen R, Borri M, Teuwen LA, Veys K, García-Caballero M, Geldhof V, *et al* (2021) Protocols for endothelial cell isolation from mouse tissues: kidney, spleen, and testis. *STAR Protoc* 2

Guidotti LG, Inverso D, Sironi L, Di Lucia P, Fioravanti J, Ganzer L, Fiocchi A, Vacca M, Aiolfi R, Sammicheli S, *et al* (2015) Immunosurveillance of the liver by intravascular effector CD8+ T cells. *Cell* 161: 486–500

Kalucka J, de Rooij LPMH, Goveia J, Rohlenova K, Dumas SJ, Meta E, Conchinha N V., Taverna F, Teuwen LA, Veys K, *et al* (2020) Single-Cell Transcriptome Atlas of Murine Endothelial Cells. *Cell* 180: 764-779.e20

Moding EJ, Clark DP, Qi Y, Li Y, Ma Y, Ghaghada K, Johnson GA, Kirsch DG & Badea CT (2013) Dual-energy micro-computed tomography imaging of radiation-induced vascular changes in primary mouse sarcomas. *Int J Radiat Oncol Biol Phys* 85: 1353–1359

Pasut A, Becker LM, Cuypers A & Carmeliet P (2021) Endothelial cell plasticity at the single-cell level. *Angiogenesis* 24: 311–326

Sano T, Kobayashi T, Negoro H, Sengiku A, Hiratsuka T, Kamioka Y, Liou LS, Ogawa O & Matsuda M (2016) Intravital imaging of mouse urothelium reveals activation of extracellular signal-regulated kinase by stretch-induced intravesical release of ATP. *Physiol Rep* 4

Smith BR, Kempen P, Bouley D, Xu A, Liu Z, Melosh N, Dai H, Sinclair R & Gambhir SS (2012) Shape matters: Intravital microscopy reveals surprising geometrical dependence for nanoparticles in tumor models of extravasation. *Nano Lett* 12: 3369–3377

Sokol L, Geldhof V, García-Caballero M, Conchinha N V., Dumas SJ, Meta E, Teuwen LA, Veys K, Chen R, Treps L, *et al* (2021) Protocols for endothelial cell isolation from mouse tissues: small intestine, colon, heart, and liver. *STAR Protoc* 2

Sorensen EW, Lian J, Ozga AJ, Miyabe Y, Ji SW, Bromley SK, Mempel TR & Luster AD (2018) CXCL10 stabilizes T cell-brain endothelial cell adhesion leading to the induction of cerebral malaria. *JCI insight* 3

Stroh M, Zimmer JP, Duda DG, Levchenko TS, Cohen KS, Brown EB, Scadden DT, Torchilin VP, Bawendi MG, Fukumura D, *et al* (2005) Quantum dots spectrally distinguish multiple species within the tumor milieu in vivo. *Nat Med* 11: 678–682

Zhao Q, Eichten A, Parveen A, Adler C, Huang Y, Wang W, Ding Y, Adler A, Nevins T, Ni M,

et al (2018) Single-cell transcriptome analyses reveal endothelial cell heterogeneity in tumors and changes following antiangiogenic treatment. *Cancer Res* 78: 2370–2382

Changes made to the Figures

- In Figure 3A the images of the control tumors were replaced with images of a different biological replicate
- In Figure 3B, the images of the whole tumors on Day 2 and 4 were replaced, because we noticed that originally we have mistakenly shown images from Day 3 and 6 instead of Day 2 and 4 for which we apologize. Additionally, we added scale bars to the magnified smaller inserts.
- In Figure 3C we added scale bars to the magnified smaller inserts.

1st Revision - Editorial Decision 8th Mar 2022

Dear Dr. Markelc

Thank you for the submission of your revised manuscript to EMBO reports. We have now received the full set of referee reports that is copied below.

As you will see, all referees are very positive about the study and request only minor changes to clarify the scope of the study in the title.

From the editorial side, there are also a few things that we need before we can proceed with the official acceptance of your study.

1) Please update the header of the Conflict of interest paragraph to 'Disclosure and competing interests statement" (see also https://www.embopress.org/competing-interests).

2) Please identify the institutional and/or licensing committee approving the experiments. Involving mice. You currently state: "All animal studies were performed in accordance with the Animals Scientific Procedure Act 1986 (UK) and local ethics review."

3) Author contributions: Please list P. Kinchesh as PKi and P. Kannan as PKa.

4) Figure callouts: We generally recommend to call out/arrange figure panels in alphabetical order and to call out all subpanels. With this in mind, we noted the following:
- Fig 3M is called out before 3A (maybe this can be rearranged?)
- Fig EV1A-H panel callouts are missing.
- Fig EV2L panel callout is missing. There is a callout to EV2R but there is no such panel.
- Appendix Fig S1A callout is missing.

5) Appendix:
- Please add page numbers to the table of content.
- Please change the nomenclature to 'Appendix Figure S#'.
- The List of Antibodies should be called 'Appendix Table S1'.
- Appendix figure legends: please add the nature of replicates (biological, technical) for Fig. S1B-D, S3.
- Appendix Fig. S1 mentions a scale bar that is not present

6) Zhao et al 2018 could be a data citation since you re-used datasets generated by these authors, if I understand correctly. In this case you can refer to both, the datasets and the study describing these. Please see our guide to authors under "Data citation" for a full description of data citations in the text and the reference list https://www.embopress.org/page/journal/14693178/authorguide#referencesformat

7) I attach here a manuscript files with a few comments and changes that I ask you to implement. Please note that the abstract should be written in present tense.

8) Please add a scale bar to the image shown in Fig. 2D

9) Finally, EMBO reports papers are accompanied online by A) a short (1-2 sentences) summary of the findings and their significance, B) 2-3 bullet points highlighting key results and C) a synopsis image that is 550x200-600 pixels large (width x height) in .png format. You can either show a model or key data in the synopsis image. Please note that the size is rather small and that text needs to be readable at the final size. Please send us this information along with the revised manuscript.

We look forward to seeing a final version of your manuscript as soon as possible.

Yours sincerely,

Martina Rembold, PhD
Senior Editor
EMBO reports

****

Referee #1:

I thank the authors for addressing all of my concerns and congratulate them on a carefully done and important study.

Referee #2:

The authors addressed my main concerns but to be in line with their responses, the title of the manuscript should include the word "mouse" and actually also refers to the tumor tissue.
It should read:
Endothelial cell death after ionizing radiation does not impair vascular structure in mouse tumor models

Referee #3:

The authors have done an adequate revision and although questions remain their study is mature and scientifically sound. I have no further comments.

Dear Dr. Markelc,

In checking your manuscript submitted to EMBO Reports it has come to our attention that the following must be addressed before we can begin the editorial process.

1. Scale bar numbers need removing from Figs 1,3,4, and EV1. This should only be mentioned in the figure legends.
Corrected.
2. Please check the figure callouts in the text. I found the following:
- Fig 2H is called out after 2K.

Corrected

Fig 4F is called out after 4D;

4P is called out after 4L;

4R is called out after 4N.

We believe these are in proper alphabetical order.

Fig 4O+S are MISSING.

Corrected

There is a callout to Fig EV2O and this panel doesn't exist in the figure.

Callout for EV2O was removed.

Fig EV3A+B are called out after EV3F.

Corrected

Figs EV4 + EV5 panel callouts are MISSING.
Corrected

3. In the Appendix please correct every mention of the figures to 'Appendix Figure S#'.
Corrected

2nd Revision - Editorial Decision 27th Jun 2022

Dr. Bostjan Markelc
University of Oxford
Department of Oncology, CRUK/MRC Oxford Institute for Radiation Oncology
Old Road Campus Research Building
Roosevelt Drive
Oxford OX3 7DQ
United Kingdom

Dear Dr. Markelc,

Thank you for implementing all editorial requests. I am now very pleased to accept your manuscript for publication in the next available issue of EMBO reports. Thank you for your contribution to our journal.

At the end of this email I include important information about how to proceed. Please ensure that you take the time to read the information and complete and return the necessary forms to allow us to publish your manuscript as quickly as possible.

As part of the EMBO publication's Transparent Editorial Process, EMBO reports publishes online a Review Process File to accompany accepted manuscripts. As you are aware, this File will be published in conjunction with your paper and will include the referee reports, your point-by-point response and all pertinent correspondence relating to the manuscript.

If you do NOT want this File to be published, please inform the editorial office within 2 days, if you have not done so already, otherwise the File will be published by default [contact: emboreports@embo.org]. If you do opt out, the Review Process File link will point to the following statement: "No Review Process File is available with this article, as the authors have chosen not to make the review process public in this case." Please note that the author checklist will still be published even if you opt out of the transparent process.

Thank you again for your contribution to EMBO reports and congratulations on a successful publication. Please consider us again in the future for your most exciting work.

Yours sincerely,

Martina Rembold, PhD
Senior Editor
EMBO reports

****

THINGS TO DO NOW:

Once your article has been received by Wiley for production, the corresponding author will receive an email from Wiley's Author Services system which will ask them to log in and will present them with the appropriate license for completion.

You will receive proofs by e-mail approximately 2-3 weeks after all relevant files have been sent to our Production Office; you should return your corrections within 2 days of receiving the proofs.

Please inform us if there is likely to be any difficulty in reaching you at the above address at that time. Failure to meet our deadlines may result in a delay of publication, or publication without your corrections.

All further communications concerning your paper should quote reference number EMBOR-2021-53221V3 and be addressed to emboreports@wiley.com.

Should you be planning a Press Release on your article, please get in contact with emboreports@wiley.com as early as possible, in order to coordinate publication and release dates.

EMBO PRESS

YOU MUST COMPLETE ALL CELLS WITH A PINK BACKGROUND ↓

PLEASE NOTE THAT THIS CHECKLIST WILL BE PUBLISHED ALONGSIDE YOUR PAPER

Corresponding Author Name: Bostjan Markelc, PhD	
Journal Submitted to: EMBO reports	
Manuscript Number: EMBOR-2021-53221	

USEFUL LINKS FOR COMPLETING THIS FORM

http://www.antibodypedia.com
http://1degreebio.org
http://www.equator-network.org/reporting-guidelines/improving-bioscience-research-repor
http://grants.nih.gov/grants/olaw/olaw.htm
http://www.mrc.ac.uk/Ourresearch/Ethicsresearchguidance/Useofanimals/index.htm
http://ClinicalTrials.gov
http://www.consort-statement.org
http://www.consort-statement.org/checklists/view/32-consort/66-title
http://www.equator-network.org/reporting-guidelines/reporting-recommendations-for-tumo
http://datadryad.org
http://figshare.com
http://www.ncbi.nlm.nih.gov/gap
http://www.ebi.ac.uk/ega
http://biomodels.net/
http://biomodels.net/miriam/
http://jjj.biochem.sun.ac.za
https://osp.od.nih.gov/biosafety-biosecurity-and-emerging-biotechnology/
http://www.selectagents.gov/

Reporting Checklist For Life Sciences Articles (Rev. June 2017)

This checklist is used to ensure good reporting standards and to improve the reproducibility of published results. These guidelines are consistent with the Principles and Guidelines for Reporting Preclinical Research issued by the NIH in 2014. Please follow the journal's authorship guidelines in preparing your manuscript.

A- Figures
1. Data
The data shown in figures should satisfy the following conditions:

- the data were obtained and processed according to the field's best practice and are presented to reflect the results of the experiments in an accurate and unbiased manner.
- figure panels include only data points, measurements or observations that can be compared to each other in a scientifically meaningful way.
- graphs include clearly labeled error bars for independent experiments and sample sizes. Unless justified, error bars should not be shown for technical replicates.
- if n< 5, the individual data points from each experiment should be plotted and any statistical test employed should be justified
- Source Data should be included to report the data underlying graphs. Please follow the guidelines set out in the author ship guidelines on Data Presentation.

2. Captions

Each figure caption should contain the following information, for each panel where they are relevant:

- a specification of the experimental system investigated (eg cell line, species name).
- the assay(s) and method(s) used to carry out the reported observations and measurements
- an explicit mention of the biological and chemical entity(ies) that are being measured.
- an explicit mention of the biological and chemical entity(ies) that are altered/varied/perturbed in a controlled manner.
- the exact sample size (n) for each experimental group/condition, given as a number, not a range;
- a description of the sample collection allowing the reader to understand whether the samples represent technical or biological replicates (including how many animals, litters, cultures, etc.).
- a statement of how many times the experiment shown was independently replicated in the laboratory.
- definitions of statistical methods and measures:
  - common tests, such as t-test (please specify whether paired vs. unpaired), simple χ2 tests, Wilcoxon and Mann-Whitney tests, can be unambiguously identified by name only, but more complex techniques should be described in the methods section;
  - are tests one-sided or two-sided?
  - are there adjustments for multiple comparisons?
  - exact statistical test results, e.g., P values = x but not P values < x;
  - definition of 'center values' as median or average;
  - definition of error bars as s.d. or s.e.m.

Any descriptions too long for the figure legend should be included in the methods section and/or with the source data.

In the pink boxes below, please ensure that the answers to the following questions are reported in the manuscript itself. Every question should be answered. If the question is not relevant to your research, please write NA (non applicable). We encourage you to include a specific subsection in the methods section for statistics, reagents, animal models and human subjects.

B- Statistics and general methods

Please fill out these boxes ↓ (Do not worry if you cannot see all your text once you press return)

1.a. How was the sample size chosen to ensure adequate power to detect a pre-specified effect size?	No sample size calculations were performed. Sample size was determined based on our previous experiences with intravital imaging.
1.b. For animal studies, include a statement about sample size estimate even if no statistical methods were used.	No sample size calculations were performed. Sample size was determined based on our previous experiences with intravital imaging.
2. Describe inclusion/exclusion criteria if samples or animals were excluded from the analysis. Were the criteria pre-established?	No data was excluded.
3. Were any steps taken to minimize the effects of subjective bias when allocating animals/samples to treatment (e.g. randomization procedure)? If yes, please describe.	Mice were randomized to control versus treatment groups
For animal studies, include a statement about randomization even if no randomization was used.	Mice were randomized to control versus treatment groups.
4.a. Were any steps taken to minimize the effects of subjective bias during group allocation or/and when assessing results (e.g. blinding of the investigator)? If yes please describe.	The investigators were not blinded to allocation during experiments and outcome assessment. Due to the nature of the treatment (irradiation) the investigators couldn't be blinded.
4.b. For animal studies, include a statement about blinding even if no blinding was done	The investigators were not blinded to allocation during experiments and outcome assessment. Due to the nature of the treatment (irradiation) the investigators couldn't be blinded.
5. For every figure, are statistical tests justified as appropriate?	Significance tests were performed using the one-way ANOVA for experiments with more than 2 conditions followed by post hoc Dunnett's or Holm-Sidak multiple comparison tests. For experiments with 2 conditions, two-tailed t-test was used. *P <0.05, **P <0.01, ***P <0.0001
Do the data meet the assumptions of the tests (e.g., normal distribution)? Describe any methods used to assess it.	To performe the One-Way ANOVA test we verified three assumptions: 1. Assumption of independence (Our experimental plan includes the independence criteria)-2. Assumption of homogeneity (Bartlett's and Brown-forsythe tests)-3.Assumption of normality (Shapiro -Wilk test). For cases where data did not meet these requirements, we used non parametrical statistics (MannWhitney tests).
Is there an estimate of variation within each group of data?	One way ANOVA tests (performed with GraphPad Prism) includes the ANOVA table (SS, DF, MS, F).

Is the variance similar between the groups that are being statistically compared?	One way ANOVA tests (performed with GraphPad Prism) includes the Brown-Forsythe test and Bartlett's test. T-test's include F test to compare variances.

C- Reagents

6. To show that antibodies were profiled for use in the system under study (assay and species), provide a citation, catalog number and/or clone number, supplementary information or reference to an antibody validation profile. e.g., Antibodypedia (see link list at top right), 1DegreeBio (see link list at top right).	All details about the antibodies used (including catalouge numbers and manufacturers) are included in Appendix.
7. Identify the source of cell lines and report if they were recently authenticated (e.g., by STR profiling) and tested for mycoplasma contamination.	B16F10 murine melanoma cells were a gift from John L. Francis, Center for Thrombosis Research, Florida Hospital, Orlando, Florida, USA. Cells were authenticated.<br>MC-38 murine colorectal cancer cells were purchased from ATCC. ells were checked regularly using MycoAlert Mycoplasma Detection Kit (Lonza Group Ltd.) to ensure cells were negative for mycoplasma.

* for all hyperlinks, please see the table at the top right of the document

D- Animal Models

8. Report species, strain, gender, age of animals and genetic modification status where applicable. Please detail housing and husbandry conditions and the source of animals.	We used in house generated genetically modified C57Bl/6 female and male 8-12 weeks old mice. Additionally we used C57Bl/6 female mice 8-12 weeks old, purchased from Charles River Laboratories, UK. Mice were housed on IVC system on a 12h light-dark cycle, with food and water provided ad libitum.
9. For experiments involving live vertebrates, include a statement of compliance with ethical regulations and identify the committee(s) approving the experiments.	All animal studies were performed in accordance with the Animals Scientific Procedure Act 1986 (UK) and local ethics review.
10. We recommend consulting the ARRIVE guidelines (see link list at top right) (PLoS Biol. 8(6), e1000412, 2010) to ensure that other relevant aspects of animal studies are adequately reported. See author guidelines, under 'Reporting Guidelines'. See also: NIH (see link list at top right) and MRC (see link list at top right) recommendations. Please confirm compliance.	ach website was consulted to confirm compliance with the inclusion criteria.

E- Human Subjects

11. Identify the committee(s) approving the study protocol.	NA
12. Include a statement confirming that informed consent was obtained from all subjects and that the experiments conformed to the principles set out in the WMA Declaration of Helsinki and the Department of Health and Human Services Belmont Report.	NA
13. For publication of patient photos, include a statement confirming that consent to publish was obtained.	NA
14. Report any restrictions on the availability (and/or on the use) of human data or samples.	NA
15. Report the clinical trial registration number (at ClinicalTrials.gov or equivalent), where applicable.	NA
16. For phase II and III randomized controlled trials, please refer to the CONSORT flow diagram (see link list at top right) and submit the CONSORT checklist (see link list at top right) with your submission. See author guidelines, under 'Reporting Guidelines'. Please confirm you have submitted this list.	NA
17. For tumor marker prognostic studies, we recommend that you follow the REMARK reporting guidelines (see link list at top right). See author guidelines, under 'Reporting Guidelines'. Please confirm you have followed these guidelines.	NA

F- Data Accessibility

18: Provide a "Data Availability" section at the end of the Materials & Methods, listing the accession codes for data generated in this study and deposited in a public database (e.g. RNA-Seq data: Gene Expression Omnibus GSE39462, Proteomics data: PRIDE PXD000208 etc.) Please refer to our author guidelines for 'Data Deposition'.<br><br>Data deposition in a public repository is mandatory for:<br>a. Protein, DNA and RNA sequences<br>b. Macromolecular structures<br>c. Crystallographic data for small molecules<br>d. Functional genomics data<br>e. Proteomics and molecular interactions	Data availability section is included.The RNAseq data discussed in this publication have been deposited in NCBI's GeneExpression Omnibus and are accessible through GEO Series accession number GSE168481 (https://www.ncbi.nlm.nih.gov/geo/query/acc.cgi?acc=GSE168481).
19. Deposition is strongly recommended for any datasets that are central and integral to the study; please consider the journal's data policy. If no structured public repository exists for a given data type, we encourage the provision of datasets in the manuscript as a Supplementary Document (see author guidelines under 'Expanded View' or in unstructured repositories such as Dryad (see link list at top right) or Figshare (see link list at top right).	NA
20. Access to human clinical and genomic datasets should be provided with as few restrictions as possible while respecting ethical obligations to the patients and relevant medical and legal issues. If practically possible and compatible with the individual consent agreement used in the study, such data should be deposited in one of the major public access-controlled repositories such as dbGAP (see link list at top right) or EGA (see link list at top right).	NA
21. Computational models that are central and integral to a study should be shared without restrictions and provided in a machine-readable form. The relevant accession numbers or links should be provided. When possible, standardized format (SBML, CellML) should be used instead of scripts (e.g. MATLAB). Authors are strongly encouraged to follow the MIRIAM guidelines (see link list at top right) and deposit their model in a public database such as Biomodels (see link list at top right) or JWS Online (see link list at top right). If computer source code is provided with the paper, it should be deposited in a public repository or included in supplementary information.	NA

G- Dual use research of concern

22. Could your study fall under dual use research restrictions? Please check biosecurity documents (see link list at top right) and list of select agents and toxins (APHIS/CDC) (see link list at top right). According to our biosecurity guidelines, provide a statement only if it could.	NA
